# Predicting left ventricular contractile function via Gaussian process emulation in aortic-banded rats

**DOI:** 10.1098/rsta.2019.0334

**Published:** 2020-05-25

**Authors:** S. Longobardi, A. Lewalle, S. Coveney, I. Sjaastad, E. K. S. Espe, W. E. Louch, C. J. Musante, A. Sher, S. A. Niederer

**Affiliations:** 1 School of Biomedical Engineering and Imaging Sciences, King’s College London, London, UK; 2 Insigneo Institute for in-silico Medicine and Department of Computer Science, University of Sheffield, Sheffield, UK; 3 Institute for Experimental Medical Research and KG Jebsen Center for Cardiac Research, University of Oslo, Oslo, Norway; 4 Pfizer Worldwide Research, Development and Medical, Cambridge, MA, USA

**Keywords:** Gaussian process, history matching, global sensitivity analysis, three-dimensional bi-ventricular model, aortic-banded rat

## Abstract

Cardiac contraction is the result of integrated cellular, tissue and organ function. Biophysical *in silico* cardiac models offer a systematic approach for studying these multi-scale interactions. The computational cost of such models is high, due to their multi-parametric and nonlinear nature. This has so far made it difficult to perform model fitting and prevented global sensitivity analysis (GSA) studies. We propose a machine learning approach based on Gaussian process emulation of model simulations using probabilistic surrogate models, which enables model parameter inference via a Bayesian history matching (HM) technique and GSA on whole-organ mechanics. This framework is applied to model healthy and aortic-banded hypertensive rats, a commonly used animal model of heart failure disease. The obtained probabilistic surrogate models accurately predicted the left ventricular pump function (*R*^2^ = 0.92 for ejection fraction). The HM technique allowed us to fit both the control and diseased virtual bi-ventricular rat heart models to magnetic resonance imaging and literature data, with model outputs from the constrained parameter space falling within 2 SD of the respective experimental values. The GSA identified Troponin C and cross-bridge kinetics as key parameters in determining both systolic and diastolic ventricular function.

This article is part of the theme issue ‘Uncertainty quantification in cardiac and cardiovascular modelling and simulation’.

## 1. Introduction

With each beat, cardiac myocytes generate tension and relax. Cellular tension is transduced into a coordinated, global whole-heart deformation resulting in an effective, system-level pump function. The integration of cellular, tissue and organ-scale mechanisms is essential for achieving efficient transduction of work into concerted myocardial contraction and relaxation. The break down of this system of integrated mechanisms can give rise to heart failure (HF).

Cardiac biophysical models provide a useful tool for studying whole-organ contraction [1] by simulation, and can therefore be used to understand how impaired cell level function is linked with impaired organ level activity. Building a virtual representation of the real system requires tuning the cardiac model’s properties and boundary conditions to experimental data. Global sensitivity analysis (GSA) studies are also needed to determine the role of cellular, tissue and haemodynamics properties on whole-organ function.

However, the high simulation cost and the multi-scale nature of the cardiac system have limited the formal parameter estimation and sensitivity analysis for this type of model. Different techniques have been employed for global parameter inference on cardiac cell models, including gradient descent [2], genetic algorithms [3], multivariate regression [4] and Markov chain Monte Carlo (MCMC) [5]. Most of these approaches are computationally intensive, and the computation burden is even higher when going from single to multiple scales.

Surrogate models (or *emulators*) can be used to replace the computationally expensive real model with a statistical representation of it, allowing rapid predictions of the model’s output. Using emulators we can thus perform the high number of model evaluations required to estimate global sensitivities. In this study, we use Gaussian process emulators (GPEs), which provide confidence bounds on predictions and can therefore be used for Bayesian history matching (HM). Previously employed for fitting models of galaxy formation [6], infectious disease transmission [7] and, more recently, human atrial cell [8], HM has shown to be a valuable tool for global parameter inference.

To demonstrate the capacity of GPE and HM to fit a virtual heart model, we tune model mechanics-related parameters to data from control and left ventricular (LV) hypertrophied rat hearts. LV hypertrophy is induced surgically by constricting the ascending aorta over four to six weeks and represents a common pathway to HF development. Aortic-banded (AB) rats are commonly used as an experimental animal model for HF pathology [9].

We also seek to quantitatively characterize the AB rats’ impaired LV contractile function. We therefore fit the virtual bi-ventricular rat model to changes in function that we measured or were recorded by others, using HM. Finally, we perform a GSA to understand how uncertainty in cell, tissue and haemodynamics model properties affect LV function.

The present work is organized as follows. Section 2 presents the rat experimental data and describes the three-dimensional multi-scale bi-ventricular mathematical model of rat heart contraction and the developed machine learning framework based on GPE. Section 3 shows results of the emulation and fitting processes and the GSA. Section 4 provides a discussion and §5 addresses some model limitations, while §6 closes with a brief summary. Additional details can be found in the electronic supplementary material.

## 2. Methods

### (a) Experimental data

We used control and AB rat data from experimental studies conducted by Røe *et al*. [10]. Briefly, aortic banding was performed in male Wistar rats (*n* = 29), and sham-operated (SHAM) rats (*n* = 23) served as controls. *In vivo* cardiac function was characterized by echocardiography six weeks after surgery. The criterion for inclusion in the AB group was posterior wall thickening (more than 1.9 mm). A cohort of rats (*n* = 8, *n* = 15 from SHAM, AB groups, respectively) underwent an examination with magnetic resonance imaging (MRI). Rats in the AB cohort showed a preserved systolic function (ejection fraction and fractional shortening). Concentric LV hypertrophy arose, as an increase in LV mass and wall thickness was observed. Moreover, evidence of reduced peak early diastolic velocity (e’) and increased early mitral inflow velocity/mitral annular early diastolic velocity (E/e’) confirmed an impaired diastolic function in the rats. Representative cine MRI data were obtained from a SHAM and AB rat. Each image set consisted of 47 frames. A time frame halfway through diastole was chosen for segmentation purposes to approximate the stress-free configuration.

### (b) Rat heart model

The heart was modelled as described previously [11,12]. Briefly, the LV and RV anatomies were represented by a cubic Hermite mesh [13] fitted to manually segmented MRI images. Rule-based fibres orientation was defined with a transmural variation from −60^°^ (epicardium) to 80^°^ (endocardium) [12]. Electrical activation in the heart was driven by a fixed intracellular calcium (Ca^2+^) stimulus applied simultaneously throughout the ventricular tissue. Previous heart models have shown that heterogeneous activation patterns have negligible impact in the small rat heart [11]. Rat ventricular myocytes’ Ca^2+^ transient was described using the Gattoni *et al.* models [14] for SHAM and AB rats hearts at 37^°^C and 6 Hz pacing frequency. Rat cellular contraction was modelled using the Land *et al.* [11] model to simulate tension generation arising from the thin and thick filaments. This model simulates troponin C, tropomyosin and cross-bridge dynamics and predicts the tension generated by cardiac muscle in response to changes in intracellular Ca^2+^ concentration, sarcomere length and velocity. The passive material properties were modelled using the Guccione transversely isotropic incompressible constitutive equation [15], which is widely used across the cardiac modelling community [1,12,16,17]. The methods applied here are generalizable and could equally be applied to other myocardium constitutive equations (e.g. [18]). The spatial boundary conditions were defined by constraining the LV basal plane to lie perpendicular to the apex–base axis, with one mesh node on the interior LV wall fixed in all directions to stop rigid body motion. The pressure boundary conditions were defined by constraining the aortic pressure, which couples the heart with the systemic circulation via a three-element Windkessel model [19].

### (c) Mapping model parameters to organ scale features

We selected a finite number of key regulators (defined in table 1) for each of the employed sub-models to have a complete description of the heart mechanics, giving the following input parameter vector for the multi-scale model:

2.1 $$m=(p,\;ap,\;z,\;c_{1},\;ca_{50},\;k_{\text{xb}},\;k_{\text{off}},\;T_{\text{ref}}).$$

**Table 1.** Input parameters of the rat bi-ventricular heart mechanics model.

label	units	definition
*p*	kPa	end-diastolic pressure
*ap*	kPa	aortic pressure
*z*	mmHg s ml^−1^	aortic characteristic impedance in Windkessel model
*c*_1_	kPa	tissue stiffness in Guccione constitutive equation
*ca*_50_	μM	calcium sensitivity at resting sarcomere length
*k*_xb_	ms^−1^	binding rate in cross-bridge cycling equation
*k*_off_	ms^−1^	unbinding rate in Ca^2+^-Troponin C cooperative binding equation
*T*_ref_	kPa	maximal cellular force in active tension development equation

The LV function was quantified by timings and magnitudes (defined in table 2) extracted from the volume (LVV) and pressure (LVP) transients. We selected 12 relevant quantities, giving the following output feature vector for the multi-scale model:

2.2 d=(EDV, ESV, EF, IVCT, ET, IVRT, Tdiast, PeakP, Tpeak, ESP, maxdP, mindP),

where the first seven components characterize the LVV curve, and the last five components characterize the LVP curve.

**Table 2.** Output features of the rat bi-ventricular heart mechanics model.

label	units	definition	label	units	definition
EDV	μL	end-diastolic volume	Tdiast	ms	diastolic time
ESV	μL	end-systolic volume	PeakP	kPa	peak pressure
EF	%	ejection fraction	Tpeak	ms	time to peak pressure
IVCT	ms	isovolumetric contraction time	ESP	kPa	end-systolic pressure
ET	ms	ejection time	maxdP	kPa ms^−1^	max. pressure rise rate
IVRT	ms	isovolumetric relaxation time	mindP	kPa ms^−1^	max. pressure decay rate

### (d) The multi-scale model of rat heart contraction

**Figure 1.**
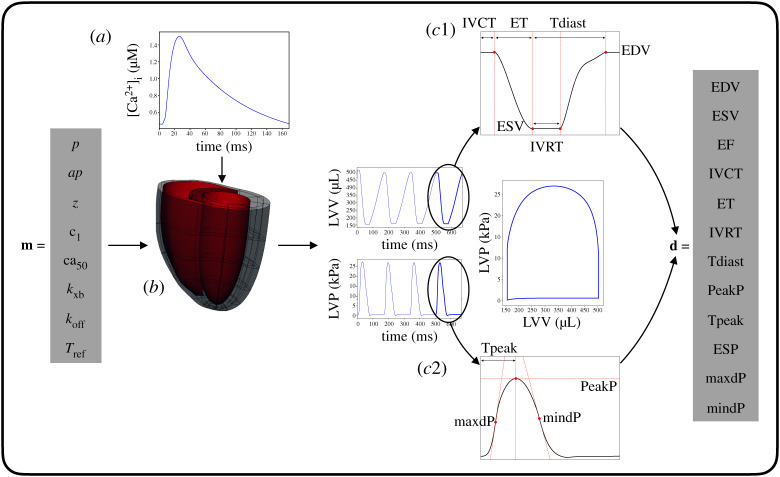
Multi-scale, bi-ventricular three-dimensional rat heart contraction model. Given a fixed calcium transient (*a*) and mesh with fibres (*b*), the vector **m** of parameters representing cell, tissue and haemodynamics properties (table 1) constitutes the input for the model. LV volume and pressure transients and pressure-volume loop are obtained after a four-beats model run. The vector **d** of LV features (table 2), extracted from the fourth-beat curves (*c*1–*c*2), constitutes the output for the model. (Online version in colour.)

Given a fixed Ca^2+^ transient and a fixed cubic Hermite template mesh with fibres as an anatomical representation, the multi-scale model of rat heart contraction can be described by a function that maps the **m** input parameter vector (equation (2.1)) into the **d** output feature vector (equation (2.2)). Figure 1 shows a schematic of the multi-scale model.

### (e) Gaussian process emulation

**Figure 2.**
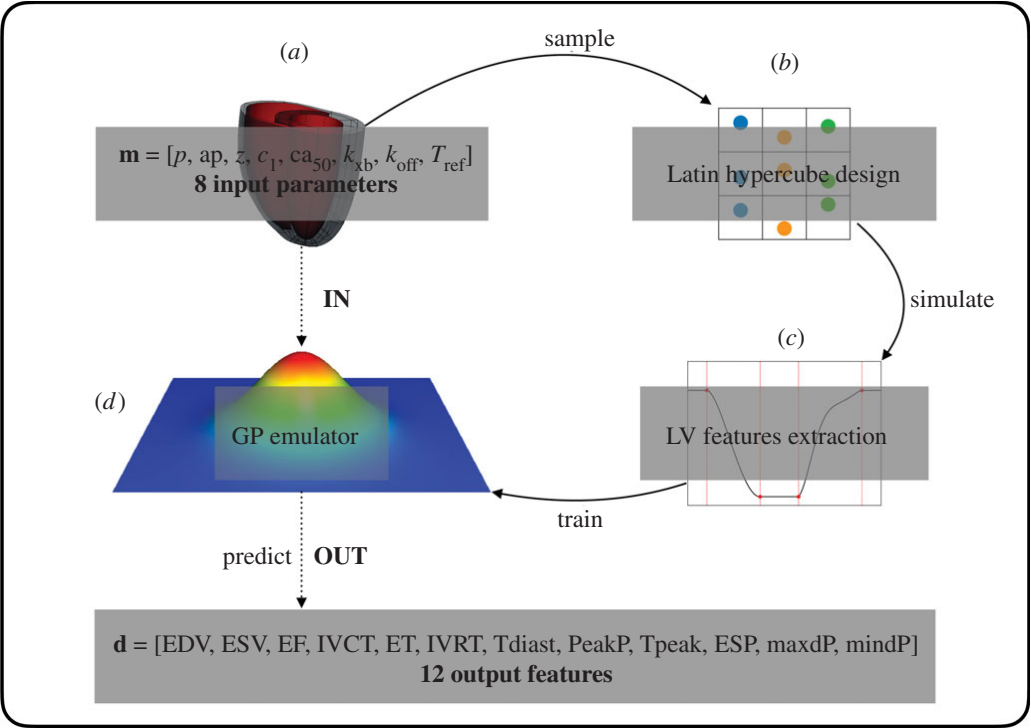
Building the surrogate model. Input points (*a*) are sampled in the parameter space using a Latin Hypercube design (*b*). The model (*simulator*) is run using these points. The simulator output is composed of features extracted from the LV pressure and volume transients (*c*). The initial input matrix and the corresponding output matrix represent the training dataset for the surrogate model (*emulator*) based on Gaussian processes. The trained emulator (*d*) is used to predict the simulator output at a new point in the input parameter space. (Online version in colour.)

The *in silico* rat heart biophysical model should aim to achieve the best representation of the real system. This requires tuning the model input parameters such that the model output features reflect the available experimental data. We trained GPEs on model simulations to replace the multi-scale model map (§2d) with a surrogate, fast evaluating map. Each GPE was defined as a combination of a mean function in the form of a linear regression model and a zero-mean GP (see electronic supplementary material, equations (1)–(3)). The GPE training is performed in two steps as described previously [6,20]. Firstly, the linear regression model’s coefficients are fitted to the data by minimizing the residual sum of squares; secondly, the GP’s hyperparameters are fitted to the residuals (data minus predicted mean) by maximization of the log-marginal likelihood function [21]. Tested mean functions were all the linear regression models (with intercept and interaction terms) with higher than first degree polynomials up to the third degree, while tested GP kernels were anisotropic covariance functions among the squared exponential kernel, Matérn_*ν*=3/2_ and Matérn_*ν*=5/2_ kernels [21]. The accuracy of a GPE was evaluated using the averaged *R*^2^ (coefficient of determination) test score obtained in a five cross-validation. Briefly, one fifth of the training dataset was excluded and the GPE was trained on the remaining points. The left-out part was then used for testing the GPE accuracy, and an *R*^2^ score was calculated. This process was repeated for each of the five subsets randomly selected from the full training dataset. The final accuracy was determined by averaging the *R*^2^ scores obtained in predicting the five different left-out parts. The developed framework is outlined in figure 2.

### (f) Target values

We constrained the model parameters based on model readouts that describe key features in the LVV and LVP transients, and pressure–volume loop (table 2). In our study, the target phenotypes’ mean and standard deviation came from two different sources. Specific organ-scale features unavailable in the MRI data [10] were deduced from literature experimental studies all performed on male rats at body temperature with abdominal/ascending aortic banding. In particular, EDV, ESV, ET and IVRT features were immediately available from the MRI data, while information about PeakP, maxdP and mindP features was collected from the literature. We did not have specific values for IVCT, Tdiast, Tpeak and ESP, and we chose not to match EF explicitly as this is derived from EDV and ESV. This gave us seven organ-scale phenotypes (see electronic supplementary material, Table S2) to constrain our model parameters.

### (g) Bayesian history matching

To constrain the initial mechanics-related parameter space to include only points that generate multi-scale models that are consistent with experimentally observed organ-scale LV features, we adopted the HM approach described in [8]. HM proceeds iteratively in ‘waves’. For each wave, the currently defined ‘not-ruled-out-yet’ (NROY) parameter space, defined by a large cloud of points, is tested with respect to an implausibility criterion: GPEs are evaluated for each NROY point, and an implausibility score reflecting the discrepancy between emulators’ predictions and data (see electronic supplementary material, equations (4)–(5)) is assigned to each point. Points with an implausibility score above a chosen ‘cutoff’ are deemed implausible, and the remaining ‘non-implausible’ subset becomes NROY for the next wave. The initial NROY space for the first wave is a large Latin hypercube design (LHD). For each following wave, the emulators are updated with data from simulations using a subset of points from the current NROY space, becoming more accurate and thus allowing NROY to be reduced further. Additional NROY points can be generated using the ‘cloud’ technique described in [8] if too few points remain for the next wave. The implausibility cutoff is reduced in each wave down to a predefined threshold, and HM continues until NROY converges.

### (h) Sobol sensitivity analysis

To assess how uncertainty in the model’s output can be attributed to different sources of uncertainty in the model input factors, we performed a variance-based sensitivity analysis. Variance-based methods of probabilistic sensitivity analysis quantify the sensitivity of the output to the model inputs in terms of a reduction in the variance of the output. We measured this reduction by estimating the Sobol sensitivity indices [22] through the Saltelli method [23] using the SALib Python library [24]. We followed the simple approach of estimating first-, second-order and total effect indices by neglecting the emulator’s uncertainty and using the emulator’s mean as a point-wise approximation of the model’s output. The employed emulators were trained on model simulations from the whole input parameter space before performing HM (first wave). Given the initial parameters’ ranges, quasi-random low-discrepancy Sobol sequences were produced and the emulators were evaluated at points belonging to these sequences in order to obtain sensitivity indices estimates.

## 3. Results

### (a) Model emulation

Eight LHDs of 1024 input parameter points each were simulated and the successfully completed simulations were collected to form the training dataset, with final size of 825 and 850 points for the SHAM and AB models, respectively. The low number of successful runs relates to a combination of failure of the mechanics simulations converging or completing a full cardiac cycle, which may happen if the contraction is insufficient to reach the aortic pressure. One GPE was trained for each output feature, for a total of 12 GPEs. The obtained cross-validation *R*^2^ test scores (GPEs’ accuracy) are reported in the electronic supplementary material, Table S1. Emulator evaluation at a new parameter set took approximately 1.2 s against a full simulator single-core run of approximately 4 h, for a total gained speedup of 12 000-fold.

The trained GPEs were firstly employed to perform an entirely *in silico* GSA. A subset of seven GPEs (specifically the ones predicting the features for which we had experimental values to match) was then used in combination with the related experimental target values to fit the rat models.

### (b) Model global sensitivity analysis

**Figure 3.**
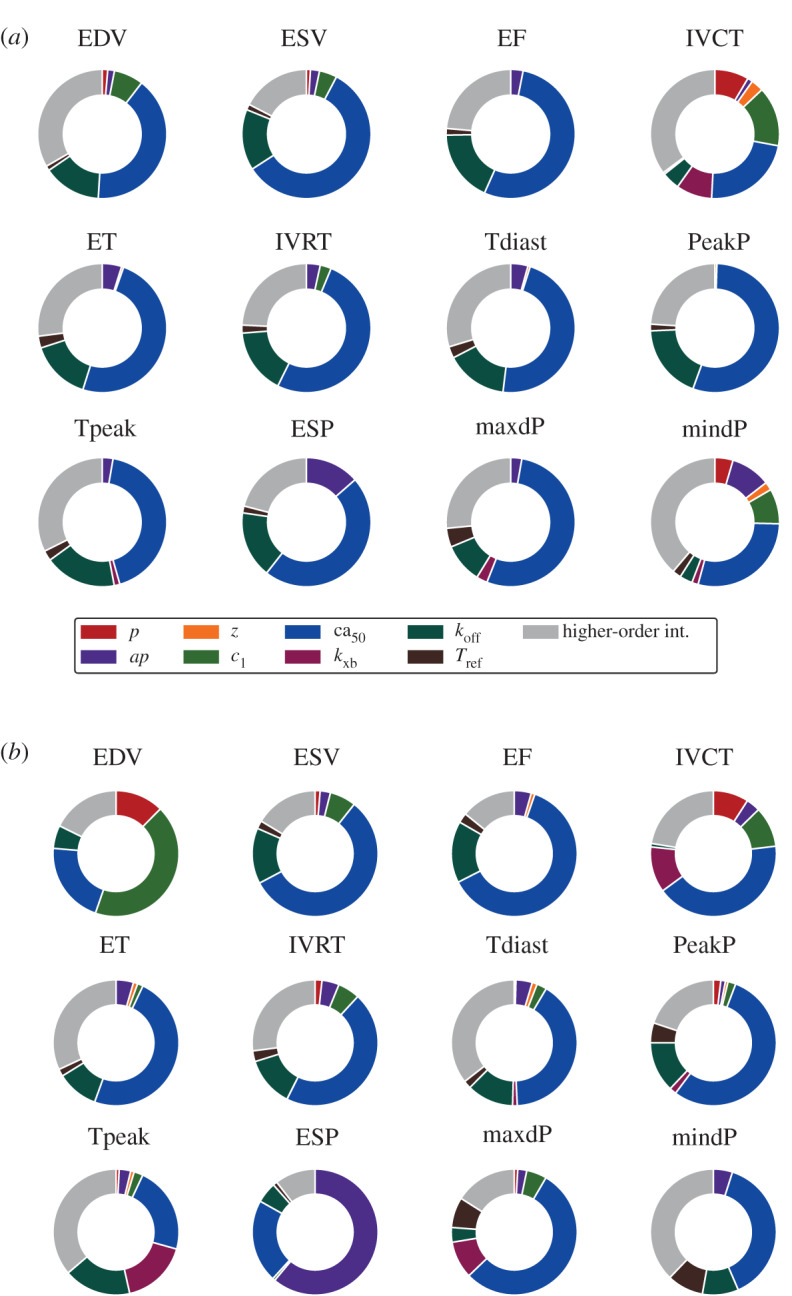
The impact of cell, tissue and boundary conditions properties on organ-scale phenotypes in SHAM (*a*) and AB (*b*) models. The contribution of each parameter is represented by the sum of its first- and (when present) second-order effects. For each LV feature, higher-order interactions (coloured in grey) are represented by the sum of total effects minus the sum of all first- and second-order effects. Parameters’ and features’ labels are explained in tables 1–2. (Online version in colour.)

Figure 3 shows how input parameters contribute to explaining each LV feature’s total variance in SHAM and AB rat models. The *ca*_50_ parameter is the most influential across all the LV features. The *k*_off_, *T*_ref_ and *ap* parameters also play a key role. All these statements are true for both rat phenotypes. By contrast, *c*_1_ is more important for AB than for SHAM, explaining a fraction of the total variance of 9 out of 12 LV features (in SHAM, *c*_1_ is important for only five features). In particular, this parameter has a significant contribution in explaining end-diastolic volume in AB (and not in SHAM). The *k*_xb_ parameter affects the AB more than the SHAM features, albeit only moderately (5 out of 12 against 4 out of 12, respectively). Finally, we observe an increased contribution of *ap* in explaining end-systolic pressure when going from SHAM to AB. A complete description of all the first- and second-order parameters’ interactions can be found in the electronic supplementary material.

### (c) Model fitting

**Figure 4.**
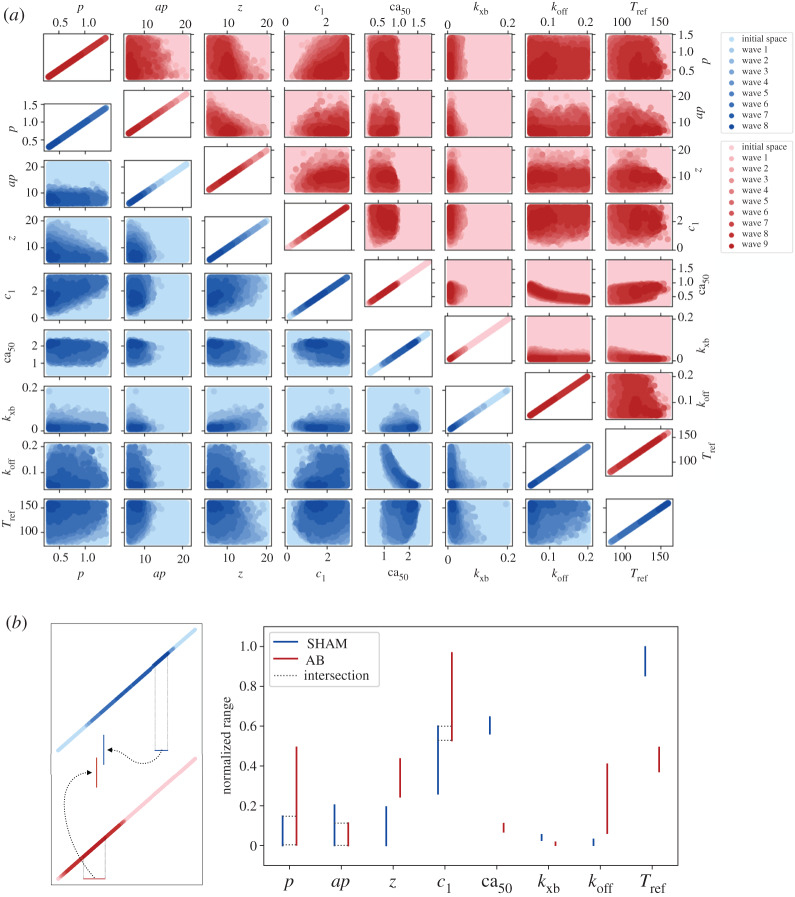
(*a*) High-dimensional input parameter space reduction during HM. SHAM HM (blue, lower diagonal axes) completed within eight waves while AB HM (red, upper diagonal axes) completed within nine waves. (*b*) Input parameters’ one-dimensional ranges after completing HM procedure. Comparison between SHAM and AB rat models’ fittings. For each input parameter, the displayed intervals are scaled according to the initial range for that specific parameter. When SHAM and AB intervals’ intersection is non-empty, this is also provided with dotted lines. Parameters’ labels are explained in table 1. (Online version in colour.)

HM is illustrated in figure 4*a* for the SHAM and AB rat models. The eight-dimensional input parameter space is plotted as a two-dimensional projection for each pair of parameters. An initial set (lightest colour variant) consisting of 400 000 points sampled with an LHD in the input parameter space was tested against an *implausibility criterion* (see electronic supplementary material, equation (5)) and became progressively constrained to smaller regions (darker colour variants) as the HM process went forward. The initial parameters’ one-dimensional ranges were determined after a literature search which included both experimental and modelling papers (see electronic supplementary material, Tables S3–S4). At each successive wave, 256 points uniformly sampled from the non-implausible region of the previous wave were simulated to build the new emulators’ training dataset along with the previously used training dataset, while the remaining points from the non-implausible region of the previous wave plus 50 000 new points sampled in the same region constituted the testing set. The threshold constant used for discerning whether or not a point was to be discarded (respectively, implausible and non-implausible point), started from 5.5 and 5.0 in the SHAM and AB models’ tuning, respectively, and was progressively reduced by 0.5 units in each wave, until reaching the commonly used value of 3.0 (e.g. [6]). The choice of these specific starting values ensured that a non-empty non-implausible set was obtained after the first wave (see the electronic supplementary material for additional information on the cutoff initial tuning). The process was terminated when the fraction of non-implausible points became small (namely 0.1% and 1.1% of the total testing set for SHAM and AB models, respectively), completing SHAM and AB models’ fitting in eight and nine waves, respectively. A total of 4352 model simulations (without considering the two initial waves, this is equivalent to a total of 15 waves × 256 points) and 1 550 000 emulator evaluations (2 LHDs × 400 000 points + 15 waves × 50 000 points) were performed.

Although HM does not ensure the reduction in individual parameters’ ranges but rather a whole high-dimensional space reduction [8], the diagonal plots in figure 4*a* suggest that all the parameters underwent reduction (to different degrees) along their one-dimensional domain, both in the SHAM and AB models. We therefore analysed whether these parameters had been constrained to lie in spaces which differ according to the rat phenotype. This phenomenon was observed in five of the eight total parameters, as highlighted in figure 4*b*. We can see that *z*, *ca*_50_, *k*_xb_, *k*_off_ and *T*_ref_ take values in completely separated spaces for the SHAM and AB models. The biggest space separation is observed for *ca*_50_ and *T*_ref_.

We finally investigated whether the constrained input parameter space was mapped by the simulator into model readouts that matched experimental observations. For this purpose, we sampled and simulated 1024 non-implausible points in the HM final wave’s parameter space of both SHAM and AB models. This was performed using rejection sampling, i.e. randomly selecting new points with components in the final wave’s reduced parameter ranges and checking their implausibility measure, eventually discarding the points with implausibility measure above the current cutoff value. Of these, only 264 and 178 points led to a successfully completed simulation for SHAM and AB models, respectively. After extracting the LV features from the simulator outputs, we examined their distributions around the related experimentally observed mean values (see electronic supplementary material, Table S2).

**Figure 5.**
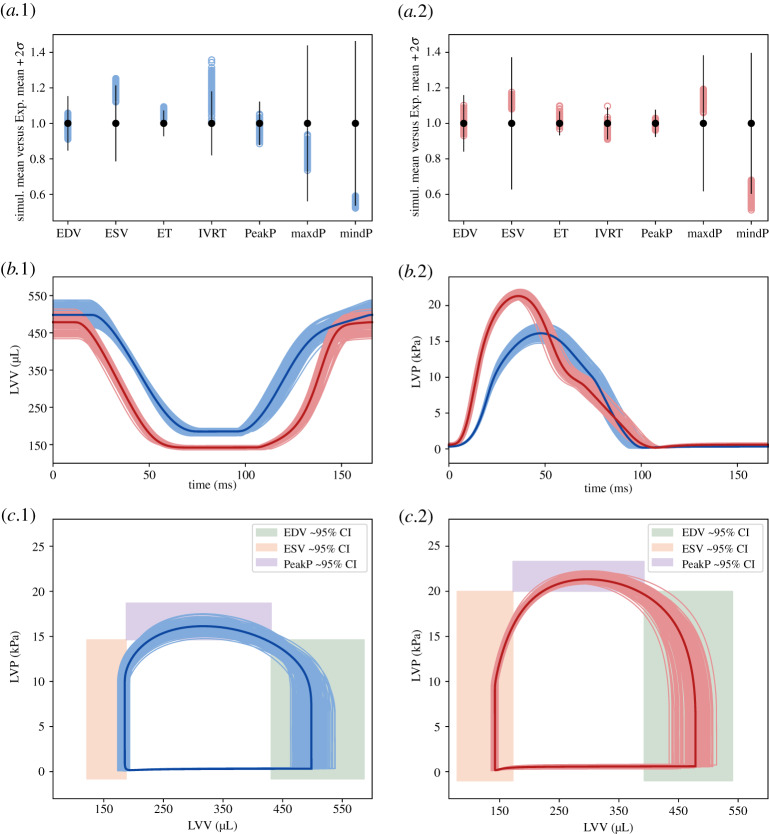
Simulator runs using the HM last wave’s parameter points as an input. (*a*) Obtained LV features’ (empty, coloured dots) distributions around experimental mean values (filled, black dots) for SHAM (.1) and AB (.2). 2 SD confidence intervals are shown as vertical straight lines centred in their respective mean value. All the displayed values (including confidence intervals) are normalized by the respective experimental mean values. Features’ labels are explained in table 2. (*b*) Simulated LV volume (.1) and pressure (.2) curves which the features in (*a*) were extracted from, for SHAM (blue) and AB (red). The average curves are displayed in darker colour variant. (*c*) Resulting SHAM (.1) and AB (.2) pressure-volume loops with approximately 95% EDV, ESV, PeakP features’ experimental confidence intervals. (Online version in colour.)

Figure 5*a*.1–2 shows that points belonging to the reduced input parameter space reproduce the majority of the emerging organ-scale LV features except ESV, maxdP and mindP which distribute far from the experimentally observed mean values. The simulated LVV and LVP transients which the features were extracted from are also reported (figure 5*b*.1–2). The resulting pressure-volume loops are provided in figure 5*c*.1–2. By comparing the highlighted average curves, we can notice smaller end-diastolic and end-systolic volumes for AB, which in turn still preserve the ejection fraction observed in the control. Diastolic-time is increased in AB due to an increase in the isovolumetric relaxation time. LV peak pressure is visibly higher in AB.

## 4. Discussion

In this study, we have shown how GPEs can be used to rapidly and robustly tune a multi-scale rat heart model to organ-scale functional measurements and characterize the global sensitivity of the model. This is the first example, to the best of our knowledge, of the use of HM to constrain multi-scale cardiac mechanics models. Previous attempts to tune cardiac mechanics model parameters have sequentially fitted parameters [25] or fitted a maximum of two to four parameters using deterministic approaches [12]. Here we fitted eight parameters concurrently. This means that error is evenly distributed across parameters and does not accumulate in the latter parameters as occurs in sequential fitting. We showed that active and passive parameters can be fitted simultaneously, avoiding potential confounding effects of slow relaxation, as occurs in HF models [16]. In addition, by providing bounds on model parameters (therefore an estimate of parameter uncertainty), the HM technique is compatible with verification, validation and uncertainty quantification (VVUQ) methods [26] supported by the ASME V&V40 standards and the FDA.^1^

HM produced different constraints for the SHAM and AB models, even if starting from the same hypercube in the input parameter space. Interestingly, the separation across the two rat phenotypes in the one-dimensional parameters’ ranges occurs mostly for parameters describing active tension development at the cellular level. We interpret this result as a confirmation that impaired whole-organ function in the AB rat is linked to altered properties at the cell level.

We have performed the first GSA of cardiac mechanics. Previous models have performed local sensitivity analysis [27], or attempted GSA on simplified cardiac cell models [28]. Our GSA approach highlighted that LV function is mostly influenced by cellular-level properties, consistent with our HM results.

## 5. Limitations

We have developed a detailed biophysical rat heart model. However, this model is inherently a simplification of the underlying system, with four specific limitations.

Firstly, the boundary conditions do not account for the pericardium that may be important in constraining cardiac mechanics [29]. We have not accounted for potential spatial variations in cellular properties and Ca^2+^ transients, and the latter homogeneously activates contraction throughout ventricular walls. Extending the sensitivity analysis to include Ca^2+^ transient features would allow us to estimate if this has a major effect on our results.

Secondly, HM provides a bounded region of non-implausible parameter sets. Parameter bounds do not define parameter distributions. Including an MCMC parameter fit using the HM bounds as priors would extend this method to estimate likely parameter distributions as opposed to parameter bounds.

The heterogeneity of the data used to constrain the models is another limitation. Specifically, pressure measurements were not available for the rats used as an animal model and were therefore collected and averaged over similar experimental studies. Related to this, we only used a single representative anatomy for the healthy rats’ cohort and single representative anatomy for the AB rats’ cohort: ejection time and isovolumetric relaxation time features were constrained according to single measurements coming from these two anatomies’ segmentations.

The fourth limitation concerns the GPE, HM and GSA implementations. In §2e, we have fitted the mean function separately and then trained the GP on the residuals, following the GPE–HM approach applied previously [20,30]. However, different emulation strategies exist (see e.g. [31], where the linear regression model parameters and the GP hyperparameters are jointly optimized; [8] uses this GP formulation for HM), and may impact the implausibility measure. To test if this choice of GP formulation impacted our final results we have preformed a preliminary comparison with the Oakley and O’Hagan [31] emulation strategy (see electronic supplementary material). This suggests that HM can potentially converge to the same reduced parameter space, however, the optimal choice of GP for fitting cardiac models requires further research.

For both HM and GSA, we used independent GPEs for each output. This univariate approach provided us the flexibility to tune the hyperparameters and choose basis functions for each output independently. However, it did not account for potential correlations in outputs, which could be accounted for with a multi-output strategy, e.g. [32], although this assumes common hyperparameters across all outputs. A multi-variate implausibility measure could be constructed from multi-output GPEs, which could potentially assist in ruling out more implausible parameter space.

In the implausibility measure (electronic supplementary material, equation (4)) calculation, we omitted the model discrepancy term, for which we did not have any estimate available. To completely replace animal models with virtual representations of them, further experts knowledge will be needed to quantify the difference between the *in silico* model and the real world system that it represents.

Finally, global sensitivity indices were estimated using the mean of the available emulator, ignoring the uncertainty of the emulator itself. However, the emulator’s uncertainty was small, therefore, the estimated sensitivity indices were still able to capture the parameters’ influence on the model outputs (see electronic supplementary material for an example on ejection fraction feature). This can be further improved in future studies by looking for alternative estimations of the sensitivity indices which directly make use of the emulator mean and variance.

## 6. Conclusion

We have shown that GPE can effectively be used to robustly and rapidly constrain multi-scale cardiac models from organ-scale measurements. The obtained virtual bi-ventricular rat hearts are a valuable system to be used for further investigation of HF. Moreover, we demonstrated that our GPE-based GSA approach enables the computationally efficient identification of key components in three-dimensional ventricular mechanics, giving a deeper insight into the link between cell, tissue, shape and boundary conditions properties and organ-scale function.

## Supplementary Material

Additional Methods and Results

## Supplementary Material

text_figures_tables.zip

## Footnotes

1 Assessing credibility of computational modeling through verification and validation.

## Data accessibility

This article has no additional data.
