# Supplementary material for: Predicting left ventricular contractile function via Gaussian process emulation in aortic-banded rats
Source: Philos Trans A Math Phys Eng Sci. 2020 May 25;378(2173):20190334. doi: 10.1098/rsta.2019.0334 (PMC7287330; doi:10.1098/rsta.2019.0334)
Supplement: Additional Methods and Results [file rsta20190334supp1.pdf]

Predicting left ventricular contractile function via Gaussian process emulation in aortic banded rats

Stefano Longobardi[1], Alexandre Lewalle[1], Sam Coveney[2], Ivar Sjaastad[3], Emil K. S. Espe[3], William E. Louch[3], Cynthia J. Musante[4], Anna Sher[4], and Steven A. Niederer[1]

[1]School of Biomedical Engineering and Imaging Sciences, King's College London, London, UK
[2]Insigneo Institute for in-silico Medicine and Department of Computer Science, University of Sheffield, Sheffield, UK
[3]Institute for Experimental Medical Research and KG Jebsen Center for Cardiac Research, University of Oslo, Oslo, Norway
[4]Pfizer Worldwide Research, Development and Medical, Cambridge, Massachusetts, USA

Supplementary material

Gaussian process emulation

The employed Gaussian process emulators (GPEs) are obtained as a combination of a mean function and a zero-mean Gaussian process (GP) (for a deeper insight on GPs, see [1]):

$$f(\mathbf{x}) := \mathbf{m}(\mathbf{x}) + \mathbf{g}(\mathbf{x}) \tag{1}$$

The mean function is of the form of a linear regression model

$$\mathbf{m}(\mathbf{x}) = \mathbf{h}(\mathbf{x})^T\boldsymbol{\beta} \tag{2}$$

while the GP is defined as

$$\mathbf{g}(\mathbf{x}) \sim \mathcal{GP}(\mathbf{0},\, k(\mathbf{x},\, \mathbf{x}')) \tag{3}$$

where $k(\mathbf{x},\, \mathbf{x}')$ is a positive definite kernel. The GPE training process consists of:

(a) fitting the linear regression model's coefficient to the data by minimising the residual sum of squares between the observed targets (data) and the targets predicted by the linear approximation;

(b) fitting the GP's hyperparameters to the residuals (data minus mean function predicted mean) by maximising the log-marginal likelihood (equation (2.30) in [1]).

Cross-validation results

LV feature		Test score					Mean test score
		**split 1**	**split 2**	**split 3**	**split 4**	**split 5**	
**EDV**	SHAM	0.9573	0.9574	0.9718	0.9685	0.9490	0.96
	AB	0.9500	0.9544	0.9235	0.9166	0.9550	0.94
**ESV**	SHAM	0.9662	0.9578	0.9648	0.9800	0.9709	0.97
	AB	0.9708	0.9544	0.9531	0.9478	0.9707	0.96
**EF**	SHAM	0.9457	0.9220	0.8787	0.9282	0.7639	0.89
	AB	0.9678	0.9517	0.9582	0.9471	0.9643	0.96
**IVCT**	SHAM	0.9699	0.9674	0.9422	0.9740	0.9584	0.96
	AB	0.9897	0.9908	0.9886	0.9894	0.9915	0.99
**ET**	SHAM	0.9242	0.8001	0.8036	0.9203	0.8436	0.86
	AB	0.9636	0.9672	0.9662	0.9301	0.9581	0.96
**IVRT**	SHAM	0.8533	0.8803	0.8940	0.9258	0.9067	0.89
	AB	0.8394	0.9082	0.8669	0.8434	0.8403	0.86
**Tdiast**	SHAM	0.9197	0.8595	0.8726	0.9528	0.8610	0.89
	AB	0.9618	0.9604	0.9554	0.9203	0.9451	0.95
**PeakP**	SHAM	0.9785	0.9721	0.9797	0.9903	0.9840	0.98
	AB	0.9827	0.9887	0.9908	0.9691	0.9882	0.98
**Tpeak**	SHAM	0.9671	0.9513	0.9614	0.9643	0.9630	0.96
	AB	0.8404	0.8891	0.9370	0.9252	0.9374	0.91
**ESP**	SHAM	0.9970	0.9971	0.9973	0.9954	0.9970	0.99
	AB	0.9958	0.9971	0.9970	0.9927	0.9959	0.99
**maxdP**	SHAM	0.9780	0.9688	0.9812	0.9672	0.9855	0.98
	AB	0.9852	0.9878	0.9910	0.9888	0.9861	0.99
**mindP**	SHAM	0.6636	0.7468	0.6824	0.8549	0.7070	0.73
	AB	0.8218	0.8543	0.6812	0.8392	0.4675	0.73

Table 1: GPEs' accuracy, evaluated using a 5-fold cross-validation. One fifth of the training dataset is excluded and one GPE is trained on the remaining points to predict each single output feature. The left-out part is then used for testing the GPE accuracy, and an R^2 score is calculated (**Test score**). The process is repeated for each of the five subsets (**split 1–5**), randomly selected from the full training dataset. The final accuracy (**Mean test score**) is determined by averaging the R^2 scores obtained in predicting the five different left-out parts. LV features' labels are explained in Table 2 in the main manuscript.

History matching formalisation

This Section provides the mathematical description of the Bayesian history matching (HM) technique (implemented following Coveney et al. [2]). HM starts by defining a dataset of observations (simulator runs). One GPE is trained for each output feature to be matched. In the first HM iteration, testing points constituting the "not-ruled-out-yet" (NROY) space are sampled from the input parameter space through a space-filling design, in our case a Latin hypercube (LHD). In the next iterations, the testing points are sampled from the NROY space of the previous iteration. A subset of NROY points is used to run simulations. The obtained simulator outputs are used to enrich the training dataset, making the GPEs more accurate in the NROY space. GPEs trained on the new space are then evaluated at the remaining points in the NROY space: if there are too few points according to the user needs (for this model: if the number of points was below 256), then additional points are generated within NROY using the "cloud" technique described in [2]. Briefly, every point in NROY space is used to generate new points by sampling from a multi-normal distribution centred on that point and scaled into the current range of the known NROY points, and then further scaled by a factor so that only 10% of the new points were non-implausible (ensuring new points are sufficiently far from the generating points).

The so called *implausibility measure* (I) is computed for each of the tested point $\mathbf{x}$ and for each output feature j:

$$I_j^2(\mathbf{x}) := \frac{(\mathbb{E}[f_{emul,j}(\mathbf{x})] - \mathbb{E}[Y_j])^2}{\mathbb{V}\text{ar}[f_{emul,j}(\mathbf{x})] + \mathbb{V}\text{ar}[Y_j] + \mathbb{V}\text{ar}[md]} \tag{4}$$

The implausibility measure takes into account how far is the emulator mean ($\mathbb{E}[f_{emul,j}(\mathbf{x})]$) from the actual observation mean ($\mathbb{E}[Y_j]$), given the observation error ($\mathbb{V}\text{ar}[Y_j]$) and the emulator uncertainty for the prediction ($\mathbb{V}\text{ar}[f_{emul,j}(\mathbf{x})]$), and an additional model discrepancy term ($\mathbb{V}\text{ar}[md]$) which in this study has not been considered.

NROY space gets eventually partitioned according to a fixed threshold into two regions: the space of non-implausible points, satisfying the inequality:

$$\max_{j \in \{1,\ldots,\#\text{output features}\}} I_j(\mathbf{x}) \leq I_{threshold} \tag{5}$$

and the space of implausible points, satisfying the reversed inequality. The non-implausible points constitute the new NROY space for the next iteration. The iterations go on while progressively scaling down the $I_{threshold}$ constant, until reaching the convergence of NROY space, i.e. the percentage of NROY space out of the total testing points space is close to zero.
The value of $I_{threshold}$ is a measure of the accepted discrepancy between the emulator and observations. This value was updated between waves. $I_{threshold}$ was manually initialised to ensure that a nonempty non-implausible set was obtained after the first wave. Starting from an $I_{threshold}$ value of 3.0 we incremented by 0.5 until we achieved a non-empty non-implausible set. This gave $I_{threshold}$ values of 5.5 and 5.0 in the SHAM and AB models' fitting, respectively. The value of $I_{threshold}$ was then decremented by 0.5 with each HM wave down to the commonly used (see e.g. [3, 4]) value of 3.0 (the so-called *Pukelsheim's 3-sigma rule* [5]). The decrement rate of 0.5 was selected to ensure that we reached the desired final value of 3.0 within a reasonable number waves, to maintain computational tractability, while allowing us to initialise the HM over a sufficient large search space.

History matching target values

This Section summarises the target organ-scale features' mean and standard deviation values for HM, which in our study come from two different sources. Whenever it was not possible to get information about a specific organ-scale feature from the available MRI data [6], the information was obtained from available literature data from experimental studies performed in male rats at body temperature with abdominal/ascending aortic banding [7, 8, 9, 10, 11, 12, 13, 14, 15]. In particular, EDV, ESV, ET and IVRT features were immediately available from the MRI data, while information about PeakP, maxdP and mindP features was collected from literature. Mean and standard deviation values for the volume-related features come from recordings performed on the cohort of rats ($n = 8$, $n = 15$ from SHAM, AB groups, respectively) that underwent MRI examination [6]. Mean and standard deviation values for each pressure-related feature were calculated from the set of experimental mean values collected from each of above-mentioned literature studies. We did not have specific values for IVCT, Tdiast, Tpeak and ESP, and we chose not to match EF explicitly as this is derived form EDV and ESV. This resulted in 7 organ-scale phenotypes (see Table 2) used to constrain the model parameters.

**Label**	**Units**	**Mean**		**Std**		**Reference**
		**SHAM**	**AB**	**SHAM**	**AB**	
EDV*	µL	508.80	466.50	39.01	37.10	[6]
ESV*	µL	154.60	125.60	16.53	23.40	[6]
ET*	ms	51.21	54.72	1.85	1.85	[6]
IVRT*	ms	20.55	41.11	1.85	1.85	[6]
PeakP**	kPa	16.66	21.66	1.02	0.69	[7, 8, 9, 10, 11, 12, 13, 14, 15]
maxdP**	$\mathrm{kPa\,ms^{-1}}$	1.14	1.24	0.25	0.24	[7, 8, 9, 10, 11, 12, 13, 14, 15]
mindP**	$\mathrm{kPa\,ms^{-1}}$	-1.00	-1.16	0.23	0.23	[7, 8, 9, 10, 11, 12, 13, 14, 15]

Table 2: Left ventricular features' target mean and standard deviation values. One asterisk $( )^*$ features values come directly from the available MRI data. Two asterisks $( )^{**}$ features values come from literature experimental studies.

History matching parameter bounds

This Section provides detailed information about the input parameters of the rat heart contraction SHAM and AB models. In the first wave of HM we used a LHD sampling of the plausible parameter space. The bounds of the plausible parameters are defined from prior experimental and modelling studies summarised in Tables 3–4. The same parameter bounds are used in both SHAM and AB models to avoid bias.

**Parameter**	**Units**	**Value from experimental papers**		**Value from modelling papers**	
		**Range**	**Ref.**	**Range**	**Ref.**
p	kPa	[0.2, 1.4]	[7, 8, 9, 10, 11, 12, 13, 14, 15]	1.0	[16, 17]
ap	kPa	[12, 21]	[7, 12, 13, 18, 19, 20, 15, 21]	[8, 9]	[16, 17]
z	$\mathrm{mmHg\,s\,ml^{-1}}$	[1.5, 16]	[22, 23, 24, 25, 26, 27, 28]	[6, 20]	[16, 17, 29]
c_1	kPa	[0.1, 3.0]	[30] (review paper)	[0.4, 1.1]	[16, 17, 31, 32]
ca_{50}	µM	–	–	[00.85, 1.56]	[16, 17, 33, 34, 35, 36]
k_{xb}	$\mathrm{ms^{-1}}$	0.1	[37, 38]	[0.008, 0.2]	[16, 17]
k_{off}	$\mathrm{ms^{-1}}$	[0.0013, 1.2]	[39, 40, 41]	[0.05, 0.2]	[16, 17, 33]
T_{ref}	kPa	–	–	[20, 160]	[16, 17, 33, 31, 37, 42, 43, 44, 45, 46]

Table 3: SHAM input parameters' ranges. The range of each parameter in HM is given by the union of experimental and modelling papers' ranges, intersected with the range resulting from the AB rat phenotype (Table 4).

**Parameter**	**Units**	**Value from experimental papers**		**Value from modelling papers**	
		**Range**	**Ref.**	**Range**	**Ref.**
p	kPa	[0.3, 1.6]	[7, 8, 9, 10, 11, 12, 13, 14, 15]	1.0	[17]
ap	kPa	[21, 26]	[7, 12, 13, 15]	[7, 20]	[17]
z	$\mathrm{mmHg\,s\,ml^{-1}}$	[5.5, 23]	[22, 26, 27]	9.5	[17]
c_1	kPa	–	–	[0.2, 1.6]	[17]
ca_{50}	µM	–	–	[00.85, 1.0]	[17]
k_{xb}	$\mathrm{ms^{-1}}$	–	–	0.02	[17]
k_{off}	$\mathrm{ms^{-1}}$	–	–	0.1	[17]
T_{ref}	kPa	–	–	120	[17]

Table 4: AB input parameters' ranges. The range for each parameter in HM is given by the union of experimental and modelling papers' ranges, intersected with the range resulting from the SHAM rat phenotype (Table 3).

Using HM technique, we fitted the SHAM and AB rat models to match the experimentally observed LV features. The input parameter space underwent reduction towards different regions according to the rat phenotype. Two different regions in the high-dimensional space were obtained even if the process started from the same hypercube for both SHAM and AB rat models. Table 5 summarises the final ranges for the mechanics-related parameters after the tuning.

**Parameter**	**Initial range**	**After fit range**	
		**SHAM**	**AB**
p	[0.3, 1.4]	[0.3, 0.5]	[0.3, 0.9]
ap	[6.0, 21.0]	[6.0, 9.1]	[6.0, 7.7]
z	[5.5, 20.0]	[5.5, 8.4]	[9.1, 11.9]
c_1	[0.1, 3.0]	[00.85, 1.9]	[1.6, 3.0]
ca_{50}	[0.4, 2.3]	[1.4, 1.7]	[0.5, 0.7]
k_{xb}	[0.008, 0.2]	[0.013, 0.019]	[0.008, 0.012]
k_{off}	[0.05, 0.2]	[0.05, 0.06]	[0.05, 0.12]
T_{ref}	[80, 160]	[148, 160]	[109, 120]

Table 5: SHAM and AB input parameters' ranges before and after the fit.

History matching verification using synthetic data

In this Section, we show the efficacy of HM technique in fitting the SHAM rat heart model to synthetic data. We started from the performed HM on the SHAM model (Figure 1). We then selected an intermediate wave, specifically wave 4 (Figure 2), among the total 8 waves the full process took to complete. We then sampled points in wave 4 (Figure 3) that did not belong to the final wave, namely wave 8. We then simulated all these points ($n = 48$) and computed the mean and standard deviation values of the observed sample for each of the organ-scale LV features, to be further used as synthetic data to be matched. We then started the HM process from the same initial space-filling design (lightest blue variant coloured points in Figure 1) used for the SHAM model HM, this time trying to match the synthetic data instead of the experimental data. To replicate exactly the same process we performed with experimental data, we tried to match the same 7 features out of the total 12 features of interest, although we had mean and standard deviation values for all the LV features. Figure 4 shows that all the LV features were perfectly matched at the end of the HM process (which took 9 waves to complete), including the 5 features we did not tried to match. Moreover, we observed that the input parameters' ranges were most of the time restricted to spaces which have nonempty intersection with the ranges where the synthetic data were sampled from (Figure 5).

**Label**	**Units**	**Mean**	**Std**
EDV*	µL	493.10	16.85
ESV*	µL	204.29	7.93
EF	%	58.54	1.74
IVCT	ms	140.855	0.78
ET*	ms	56.77	1.53
IVRT*	ms	20.03	2.43
Tdiast	ms	94.07	1.11
PeakP*	kPa	18.56	1.14
Tpeak	ms	39.39	1.36
ESP	kPa	8.74	0.21
maxdP*	kPa ms^{-1}	1.08	0.06
mindP*	kPa ms^{-1}	-0.54	0.06

Table 6: Synthetic data left ventricular features' mean and standard deviation values for the history matching. One asterisk ( )* features only were matched.

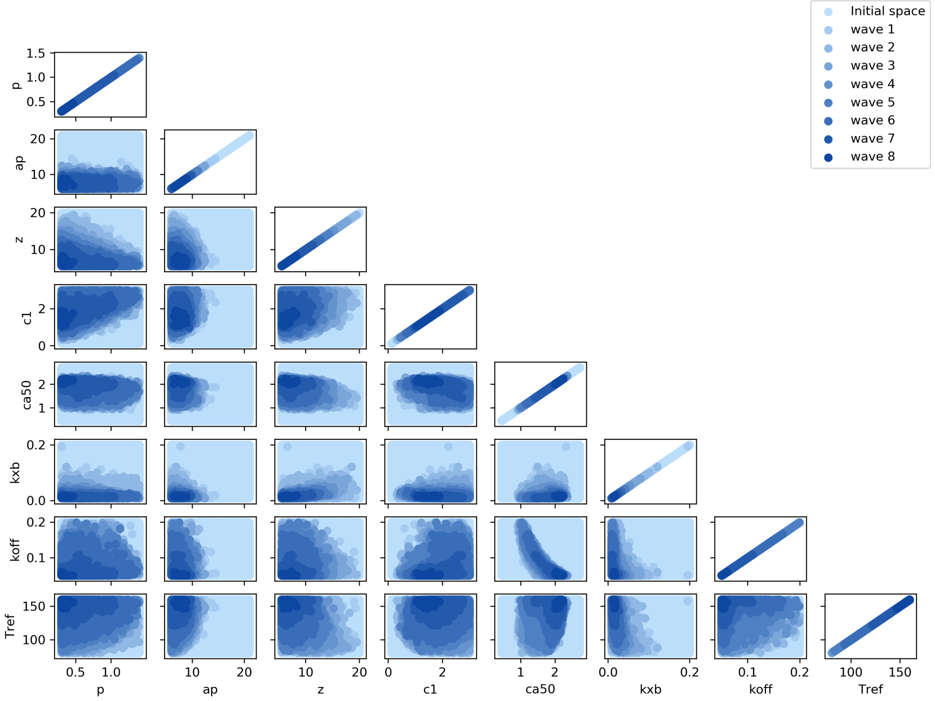

Figure 1: SHAM rat heart model history matching on experimental data.

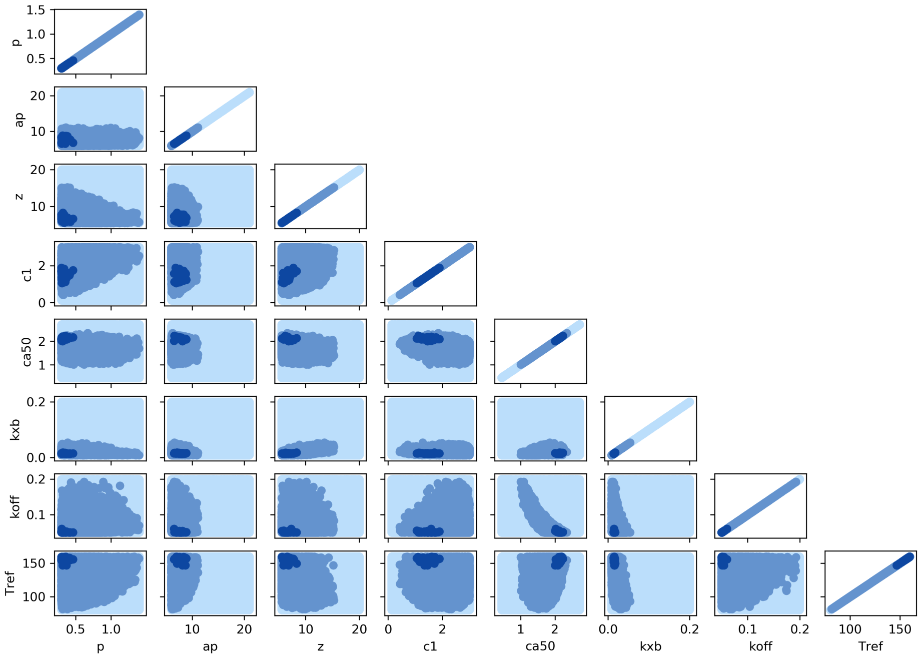

Figure 2: SHAM rat heart model history matching using experimental data. Initial space, the 4th wave NROY space and the 8th wave NROY space are highlighted with light, dark and very dark blue colour variants, respectively.

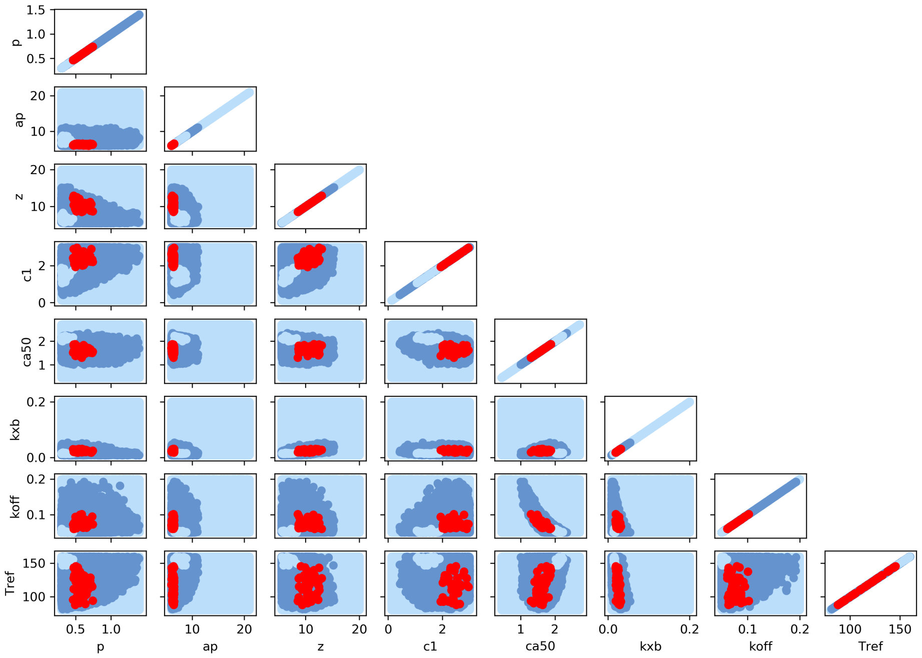

Figure 3: SHAM rat heart model history matching using experimental data. Initial space and the 4th wave space are highlighted with light and dark blue colour variants respectively. New points (in red) are sampled within the 4th wave NROY space, where the 8th wave NROY space has been cut out.

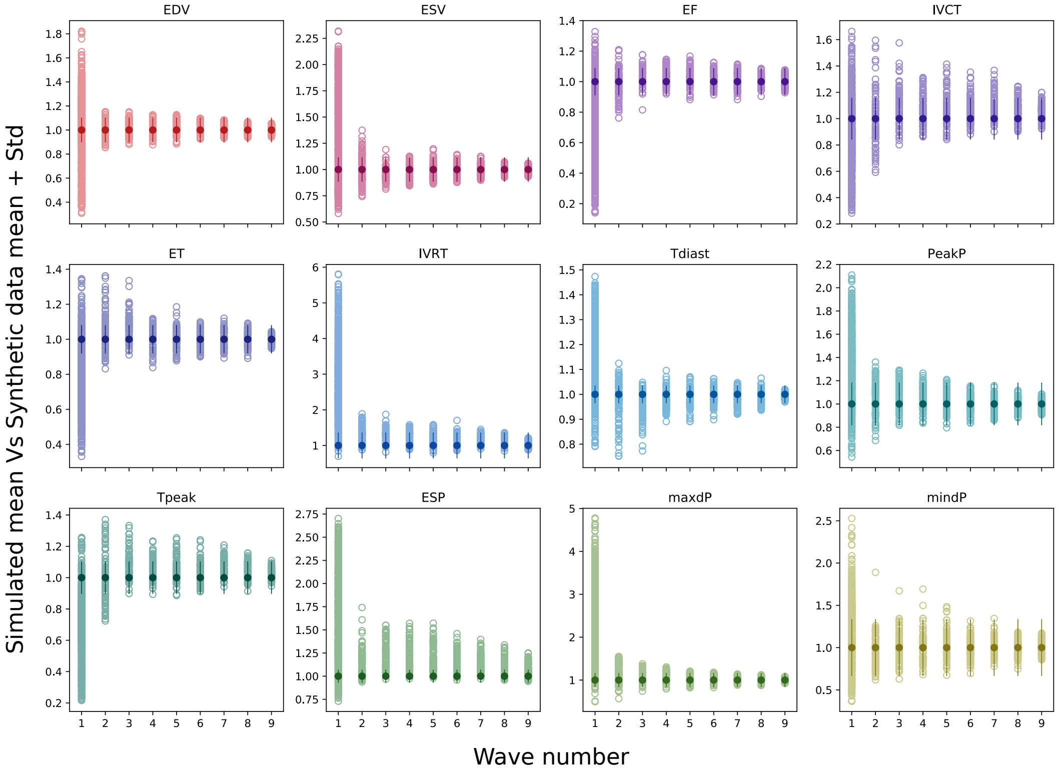

Figure 4: Simulated LV features' distributions around synthetic mean values vector. These are obtained by evaluating the simulator at input parameter points belonging to the first up to the last waves of history matching performed on synthetic data. Simulated features are shown as empty dots, while the related mean values are displayed as filled dots. 3×STD confidence intervals are also shown as vertical straight lines centred around their respective mean value. All displayed values (including confidence intervals) are normalised by their respective synthetic mean values.

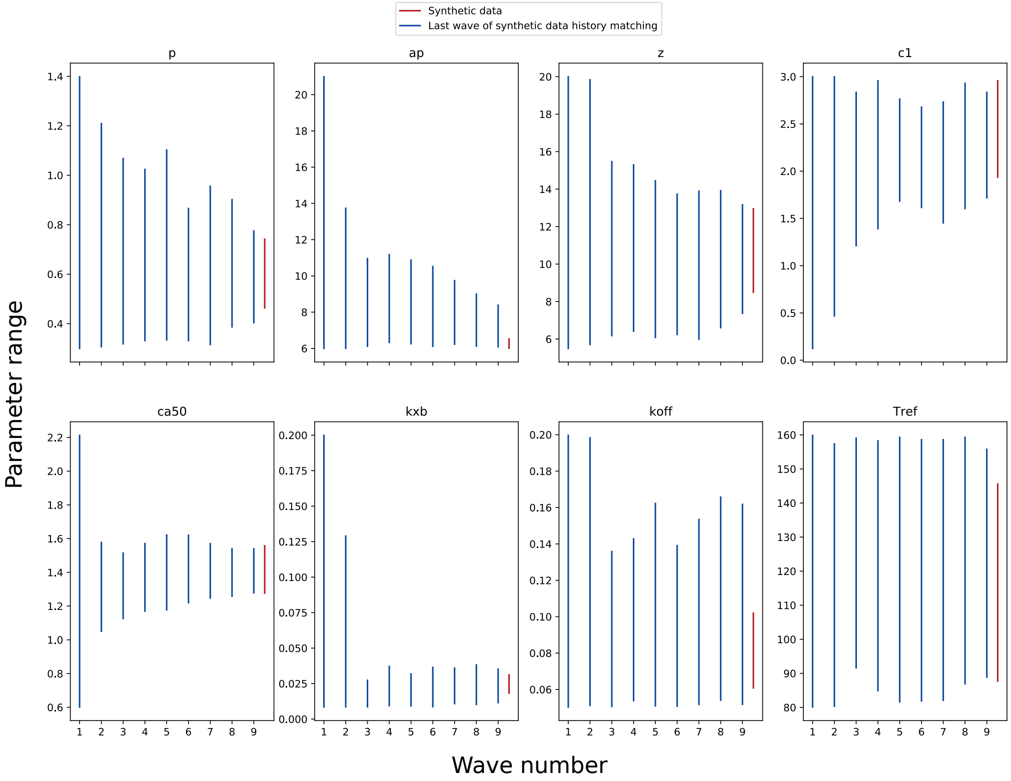

Figure 5: Input parameters' ranges at each wave during SHAM rat heart model history matching on synthetic data (blue). The parameters' ranges where the synthetic data were sampled from are also shown (red).

Investigating different emulation techniques

In Section 2(e) of the main manuscript, we used a specific formulation which describes the GPE as a combination of a mean function in the form of a linear regression model and a zero mean GP. Although this formulation served well in the context of HM (as previously shown by others, e.g. [3, 4]) being fast and easy to repeatedly train in consecutive waves, it is still a heuristic formulation and can potentially lead to underestimating the surrogate model uncertainty in the predictions. In this Section, we investigate a different emulation strategy, as presented in the work of Oakley and O'Hagan [47] and used for HM in [2]. The main difference with the currently used emulation strategy (described in the Section "Gaussian process emulation") is that the linear regressor's coefficients are concurrently fitted with the GP's hyperparameters by maximal likelihood. This approach can potentially lead to a better representation of the surrogate model uncertainty.

To investigate the possible underestimation of the emulator's variance, we evaluated how the choice of a different emulation strategy could impact the HM implausibility condition and consequent high-dimensional parameters' space reduction. From now on, we will refer to the emulators trained with the strategy employed throughout this entire work as "sequential GPEs", while we will refer to the Oakley and O'Hagan [47] emulation strategy (currently under study) as "simultaneous GPEs". The following analysis has been performed for both the SHAM and AB rat models.

We trained simultaneous GPEs (with a linear regression model and RBF kernel) using the HM initial training dataset used for the sequential GPEs (first wave). For the sake of a better visualisation, a 1 million points LHD was sampled in the initial parameter hypercube, and was tested against the implausibility criterion (equation (5)) using both the sequential GPEs and the simultaneous GPEs, with same thresholds as in the original analysis (5.5 and 5.0 for SHAM and AB, respectively). We then repeated the same process for the last wave: simultaneous GPEs were trained on the last wave's training dataset derived using the sequential GPEs. Another LHD with 1 million points was sampled this time in the second to last wave's NROY space and was tested against the implausibility criterion using both the sequential GPEs and the simultaneous GPEs, with threshold set to 3.0.

In Figure 6–7, the implausibility measures obtained by testing all the points in the first wave's LHD when using the two different emulation strategies for both of the rat models are plotted according to their magnitude in a hexbin plot. We can compare the input parameters' high-dimensional space reduction both by visual inspection and by computing the percentage of space removed in each wave 1- non-implausible (NIMP) / implausible (IMP) spaces. We can notice that, for both of the rat models, a bigger part of the space is cut using the sequential GPEs (B) compared with the simultaneous GPEs (A). For the SHAM rat the parameter space is reduced by 98.9134% and 99.9117% for the sequential and the simultaneous GPEs, respectively. For the AB rat the parameter space is reduced by 99.4345% and 99.918% for the sequential and the simultaneous GPEs, respectively.

At the last wave, however, the situation is different. At this point the accuracy of both GPEs are improved by higher sampling in the area of interest. Under these conditions, the simultaneous and sequential GPEs behave similarly. In Figure 8–9, the implausibility measures obtained by testing all the points in the last wave's LHD when using the two different emulation strategies for both of the rat models are plotted in the same manner as done in the previous case. We can notice that, for both of the rat models, the space is split into non-implausible and implausible regions in a very similar way. Again, inspecting the ratio values gives a better understanding of the modalities of the high-dimensional space splitting: 99.5202%, 99.1696% for respectively simultaneous and

sequential GPEs for SHAM, and 97.187%, 97.9942% for respectively simultaneous and sequential GPEs for AB.

We can conclude that, given the bigger space cut, there might be a non-negligible underestimation of the emulator's variance in the first wave when using the sequential GPE formulation. Smaller variances decrease the denominator in equation (4), thereby increasing the numerator and the implausibility measure for those specific points which could be therefore deemed implausible and discarded. However, with each HM wave the emulator becomes progressively more and more accurate, the underestimation of the uncertainty phenomenon is reduced and the two GPEs formulations reach the same space. This was further assessed by comparing the one-dimensional parameters' ranges we obtained using the sequential GPEs and the ones we now obtain using the simultaneous GPEs. These are summarised in Figure 10. We can see that the parameters' ranges resulting from the last wave are very similar for both of the rat models.

We have provided an initial test of how differences in GPE formulations impact HM solutions. A comprehensive evaluation is beyond the scope of this study. Different groups are using the two GPE formulations described here ([2, 4, 48, 49, 50]) and determining the costs and benefits of each approach in specific contexts would prove useful.

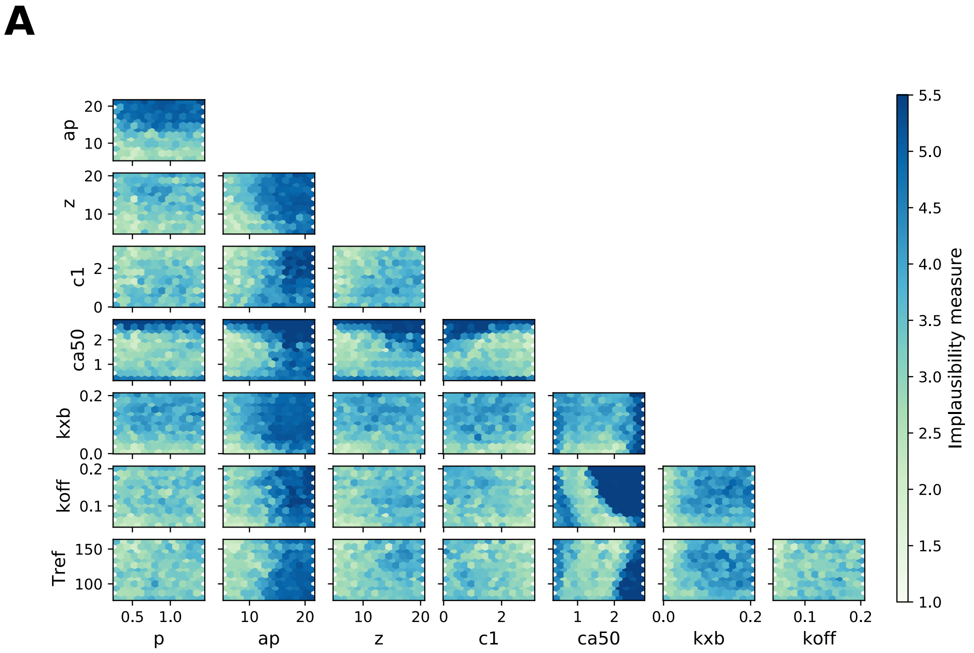

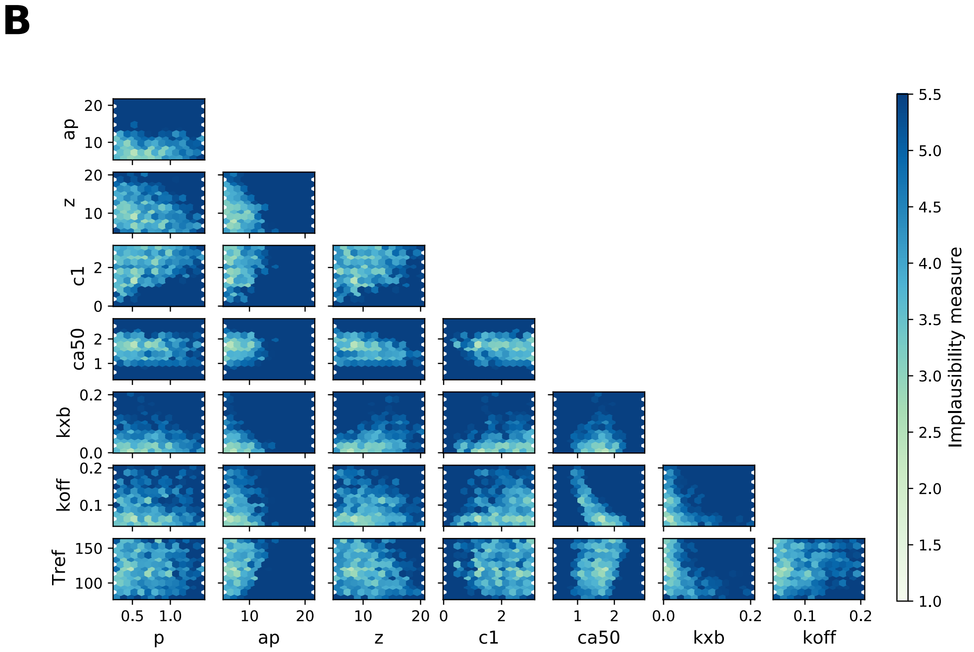

Figure 6: SHAM model, initial wave. (A) simultaneous GPEs formulation vs. (B) sequential GPEs formulation. Each hexbin is coloured by the minimum implausibility of all the points falling into it.

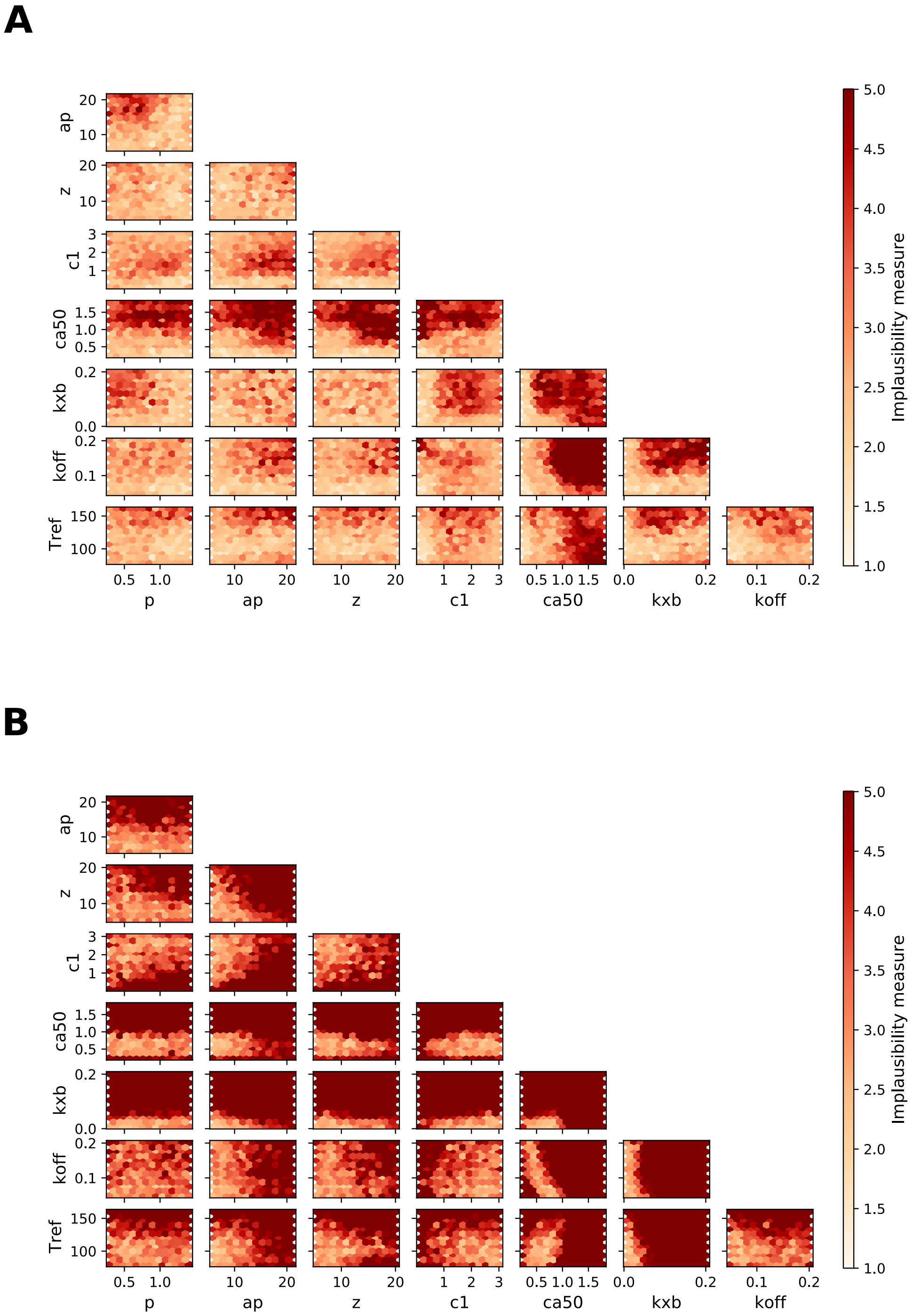

Figure 7: AB model, initial wave. (A) simultaneous GPEs formulation vs. (B) sequential GPEs formulation. Each hexbin is coloured by the minimum implausibility of all the points falling into it.

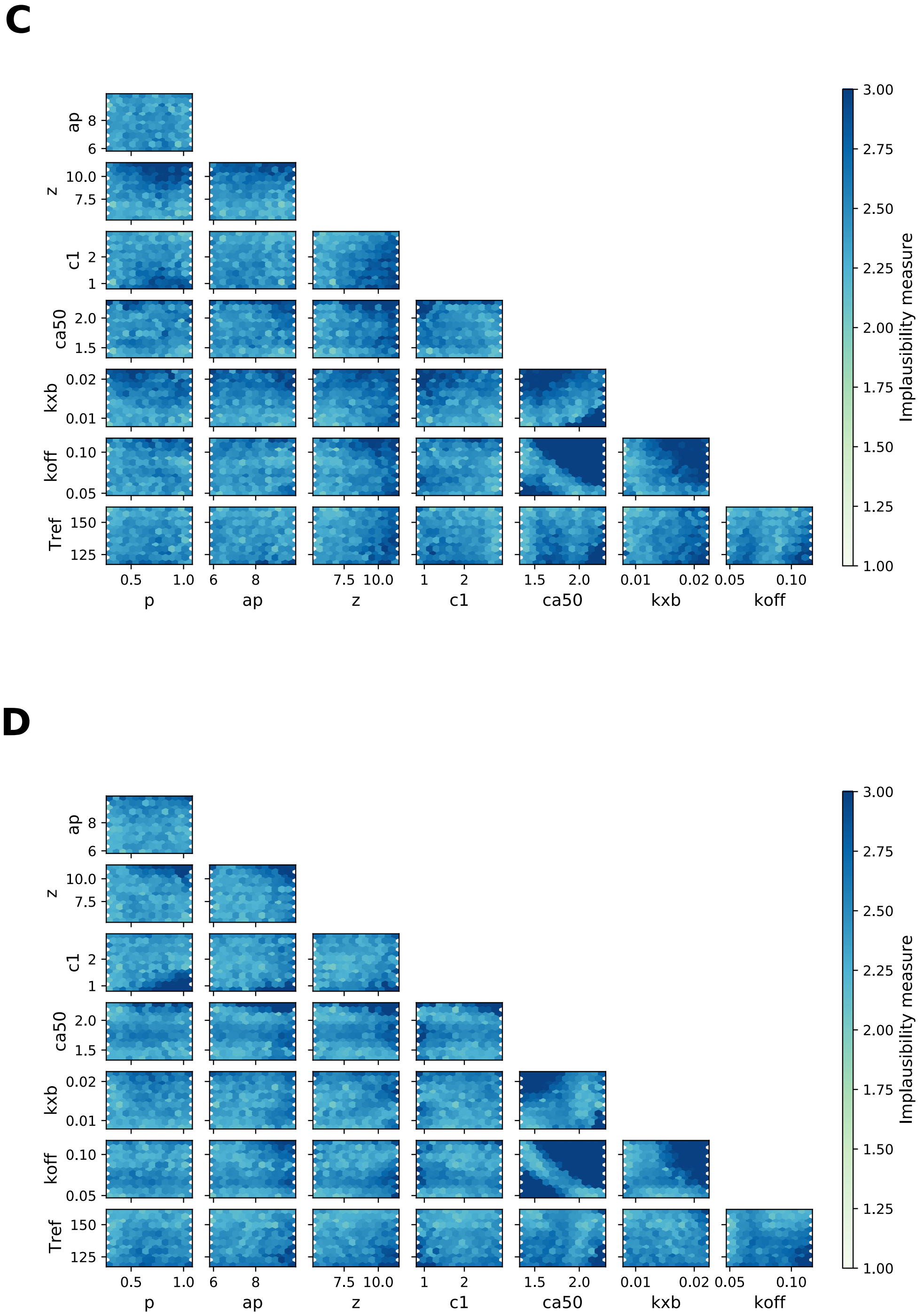

Figure 8: SHAM model, final wave. (C) simultaneous GPEs formulation vs. (D) sequential GPEs formulation. Each hexbin is coloured by the minimum implausibility of all the points falling into it.

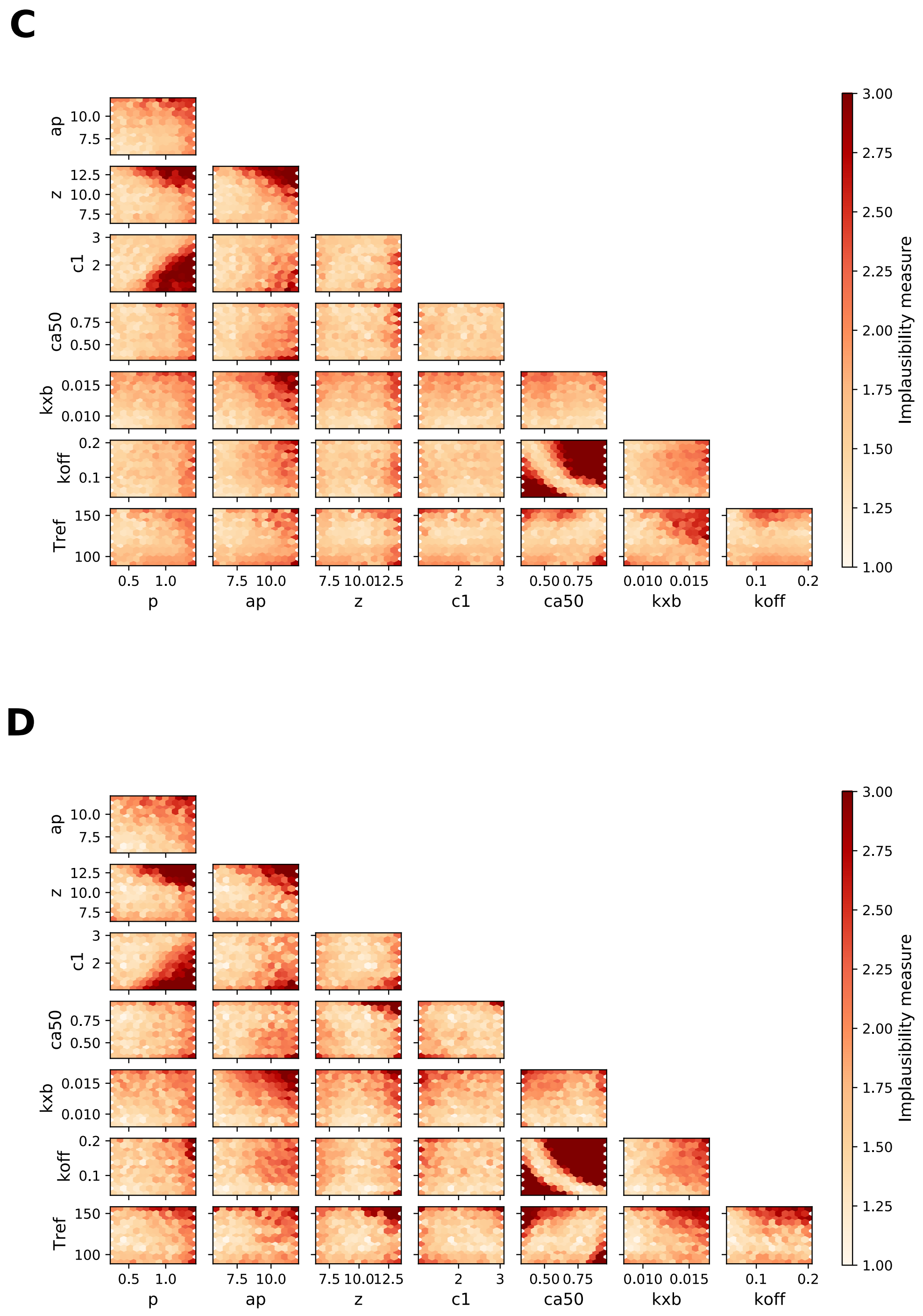

Figure 9: AB model, final wave. (C) simultaneous GPEs formulation vs. (D) sequential GPEs formulation. Each hexbin is coloured by the minimum implausibility of all the points falling into it.

A

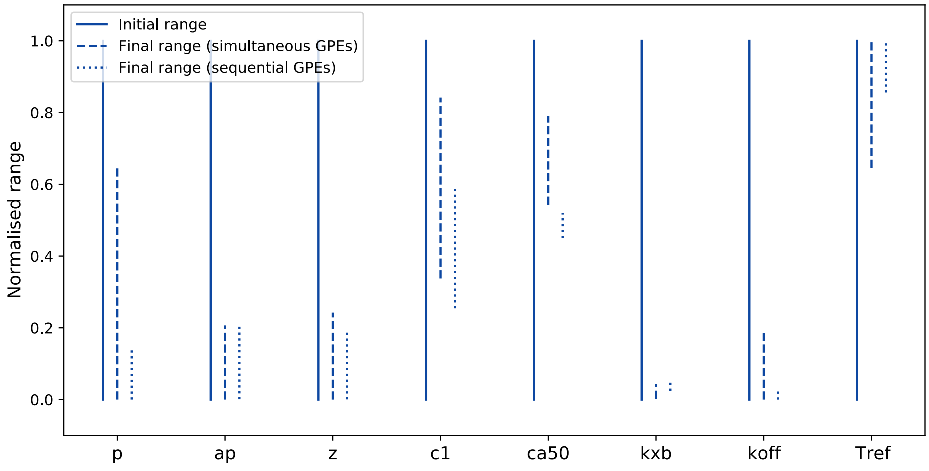

B

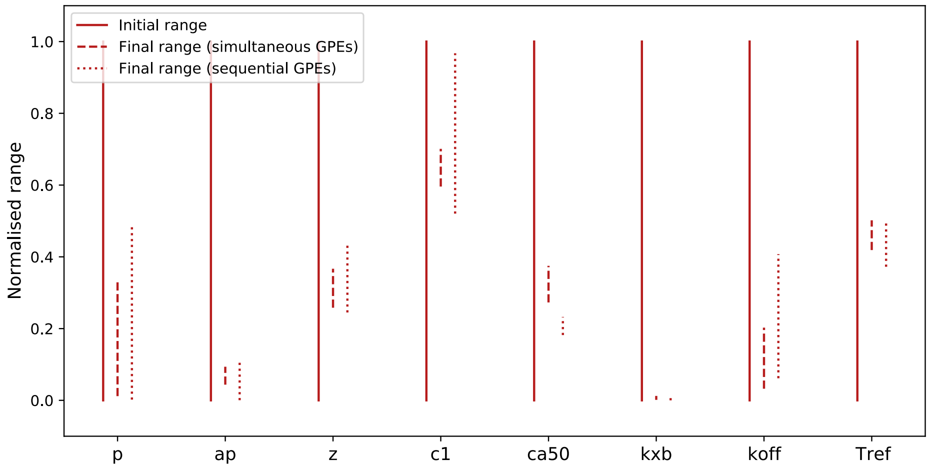

Figure 10: Final wave one-dimension parameters' ranges simultaneous GPEs vs. sequential GPEs comparison. (A) SHAM rat model. (B) AB rat model. For each parameter, the final ranges (dashed and dotted lines) have been normalised according to the relative initial range (solid line).

Global Sensitivity Analysis

This Section summarises the GP emulators-based global sensitivity analysis results. The first- and second-order interactions and total effects of input parameters for each of the organ-scale LV feature under study are shown in Figures 12–15. Figure 11 provides an example on how to read the network charts in Figures 12–15. Figure 11, for example, illustrates that the most significant second-order interaction is given by the ca_{50}–k_{off} pair of parameters.

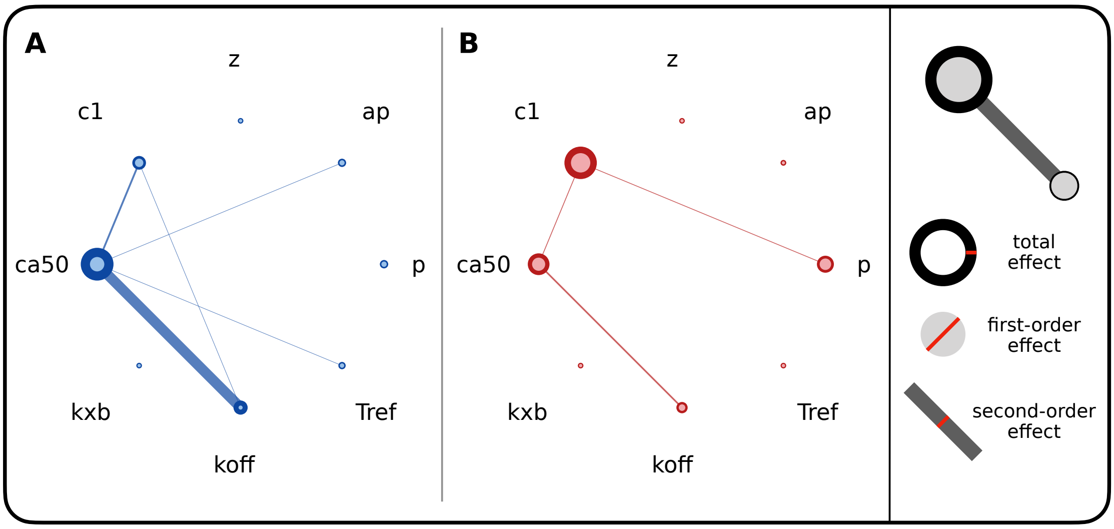

Figure 11: Example network charts. The two charts illustrate input parameters' contribution to a specific LV feature's total variance in SHAM (A) and AB (B) models. For each input parameter, the size of the node represents the first-order effect, the thickness of the linking line represents the second-order effect, and the node border thickness represents the total effect.

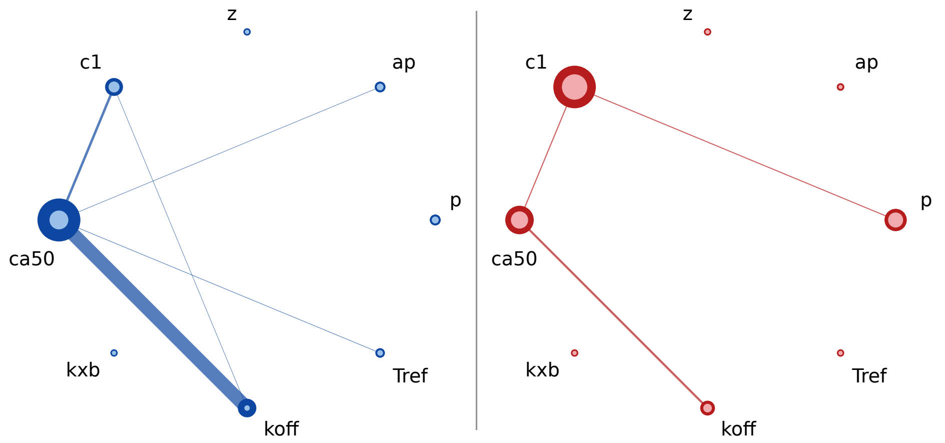

(a) EDV

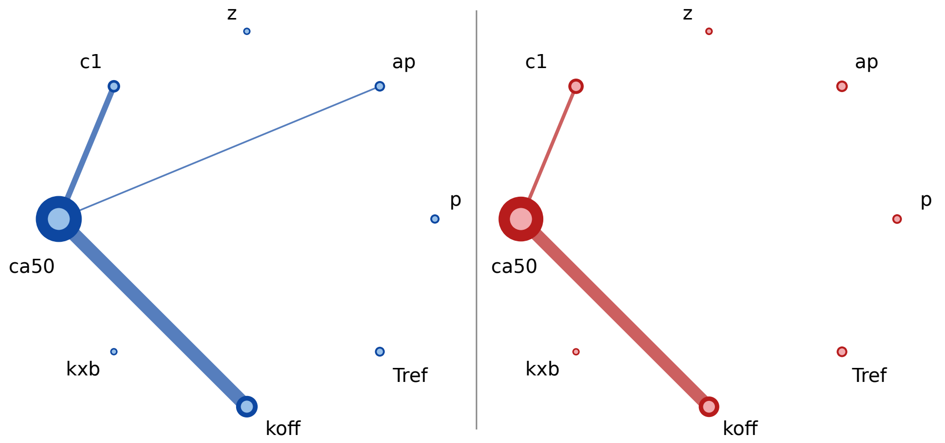

(b) ESV

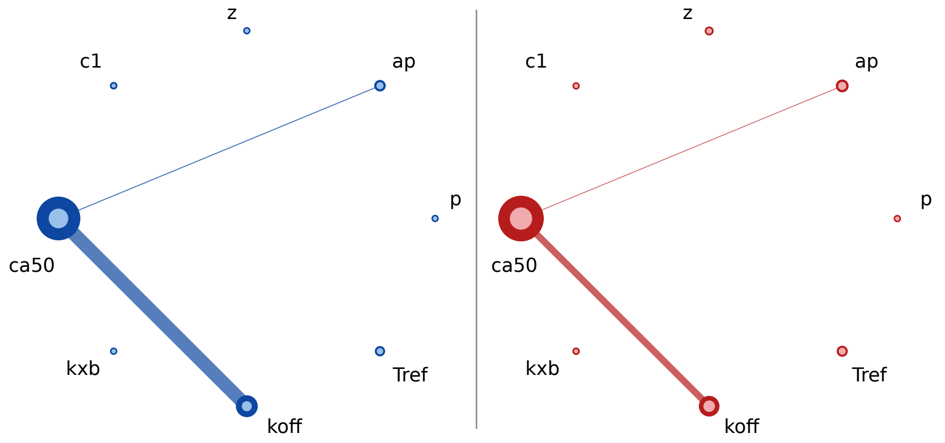

(c) EF

Figure 12: Effect of input parameters on the EDV (a), ESV (b), and EF (c) features in SHAM (blue) and AB (red) models.

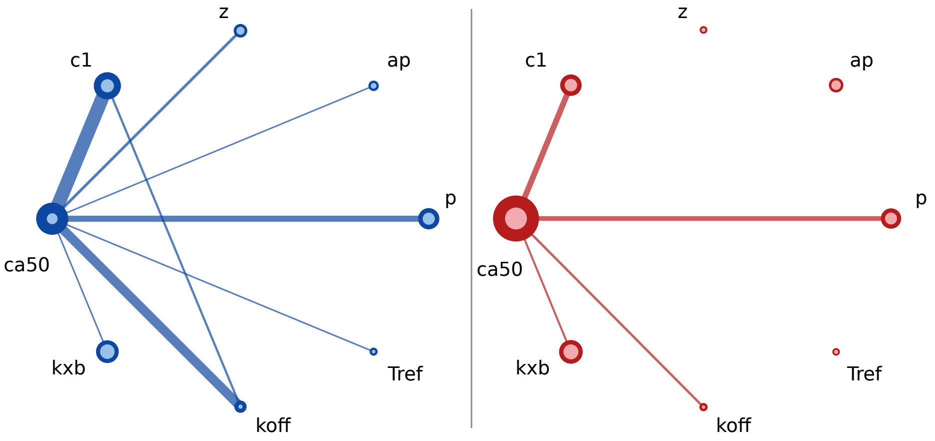

(a) **IVCT**

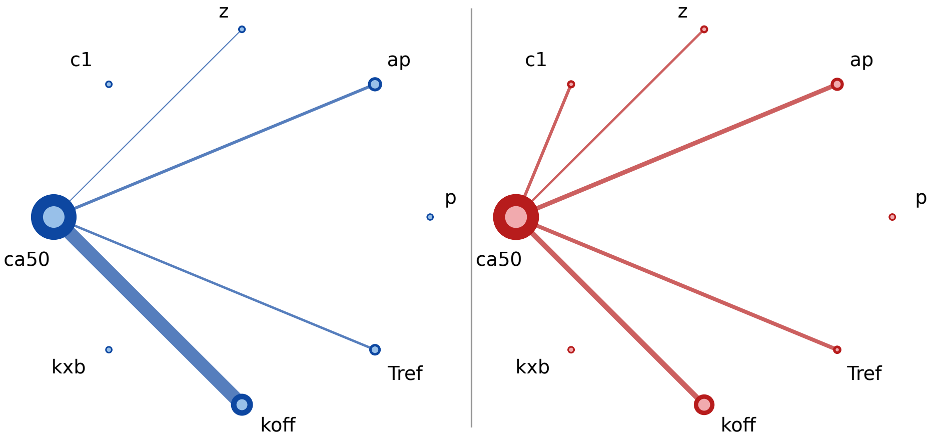

(b) **ET**

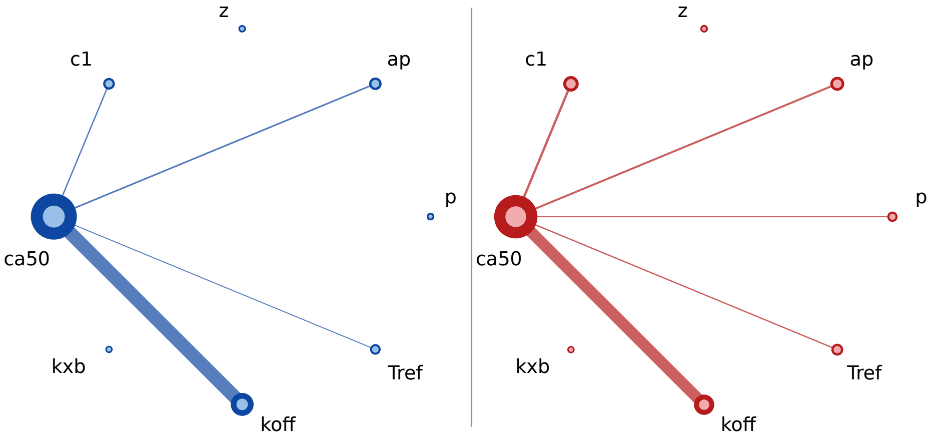

(c) **IVRT**

Figure 13: Effect of input parameters on the IVCT (a), ET (b), and IVRT (c) features in SHAM (blue) and AB (red) models.

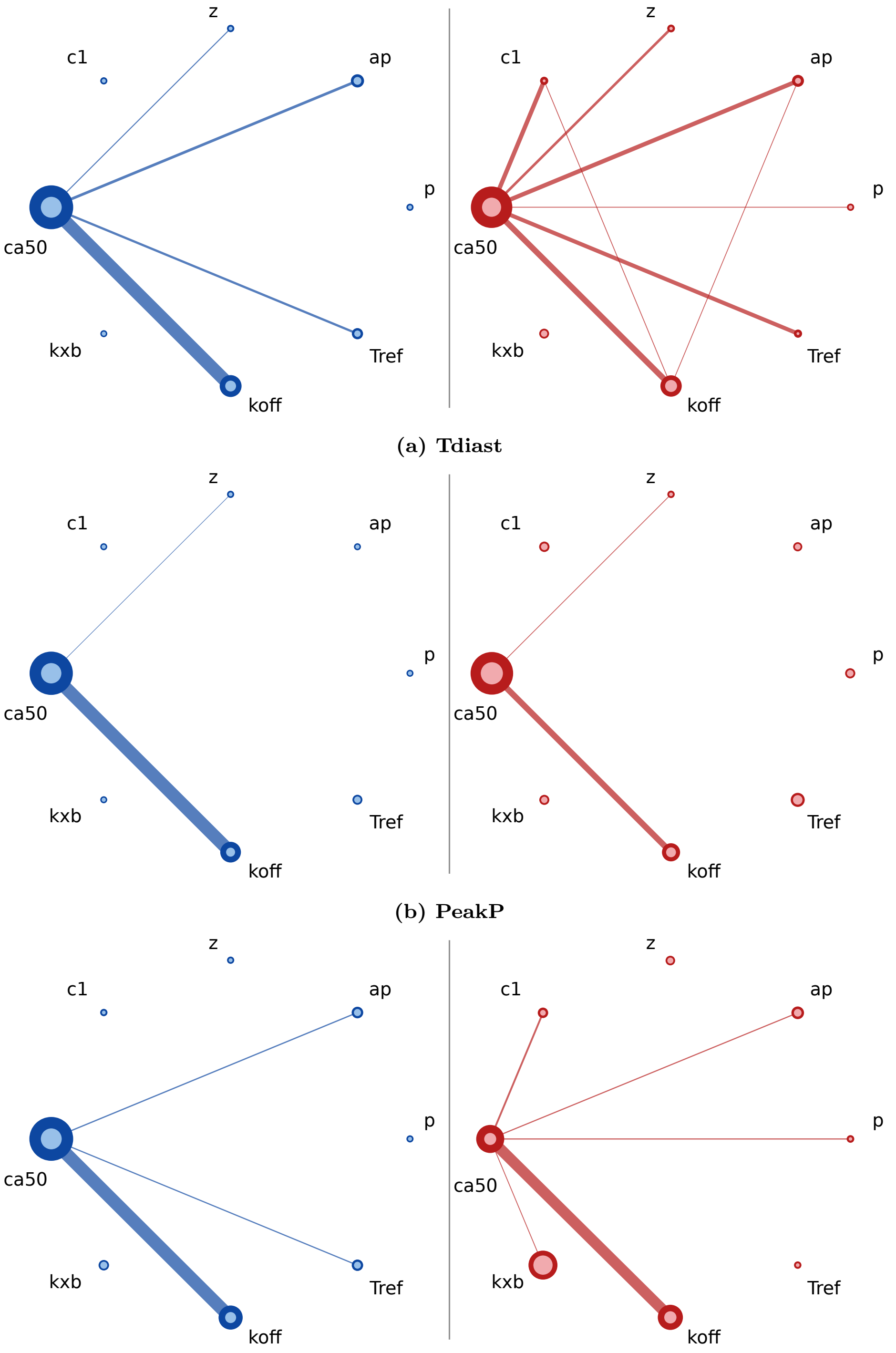

(a) **Tdiast**

(b) **PeakP**

(c) **Tpeak**

Figure 14: Effect of input parameters on the Tdiast (a), PeakP (b), and Tpeak (c) features in SHAM (blue) and AB (red) models.

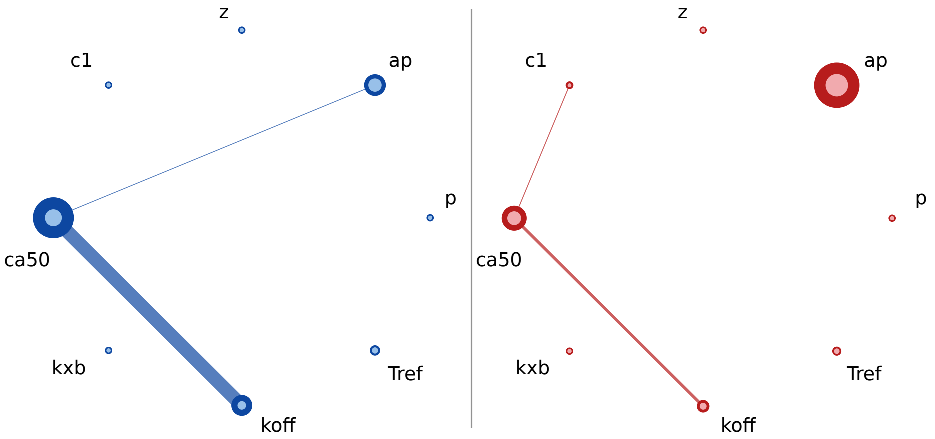

(a) ESP

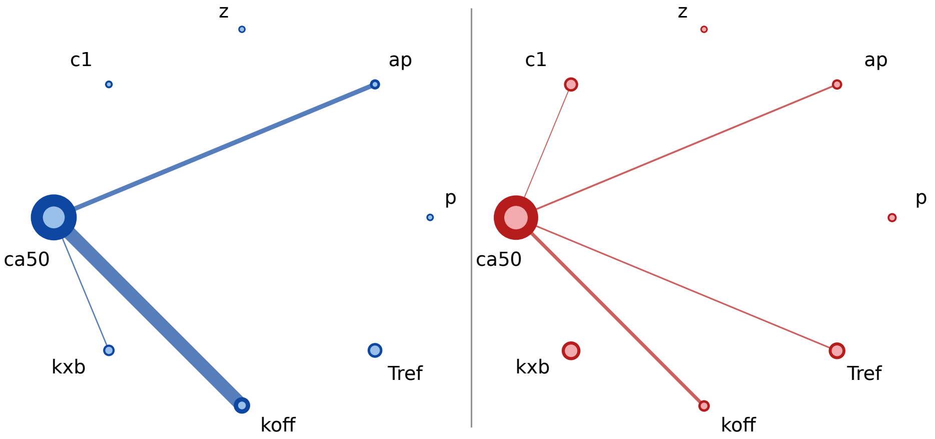

(b) maxdP

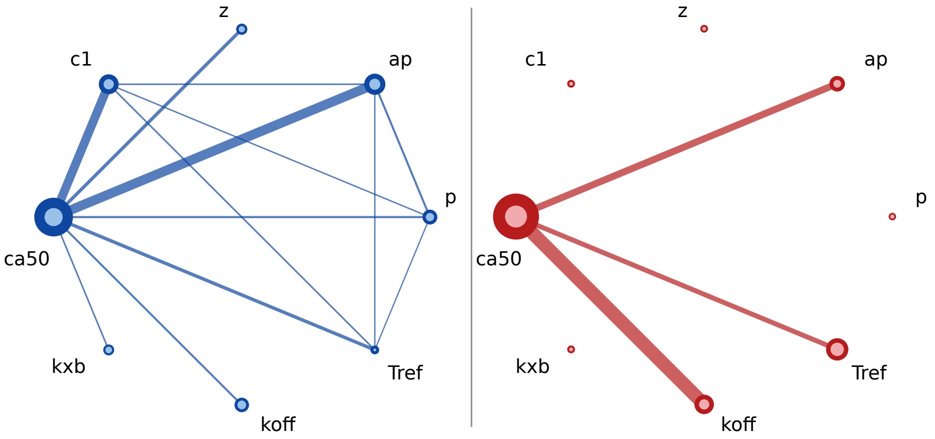

(c) mindP

Figure 15: Effect of input parameters on the ESP (a), maxdP (b), and mindP (c) features in SHAM (blue) and AB (red) models.

Sensitivity indices estimation accounting for emulator uncertainty

In Section 2(h) of the main manuscript, we mentioned that global sensitivity indices were estimated by neglecting the emulator's uncertainty and using the emulator's mean as a pointwise approximation of the model's output. This was addressed as a model limitation (discussed in Section 5) and partially motivated by the fact the emulator uncertainty around the predictions was small.

In this Section, we provide additional information to show that this choice does not affect the final sensitivity indices estimates.

The Sobol sensitivity indices were estimated using Saltelli method [51] which efficiently estimates first- and second-order and total effects using full model evaluations at a specific set of points belonging to low-discrepancy quasi-random Sobol sequences. Without going into details, let's suppose that the Saltelli estimator can be described by a function $\mathcal{S}$ that takes as an input model f evaluations Y at specific points X ($Y = f(X)$) and gives as an output the sensitivity indices S_i, S_{ij}, S_T:

$$Y \xmapsto{\mathcal{S}(\cdot)} S_i,\, S_{ij},\, S_T \tag{6}$$

In this study, we substituted the full model f with a surrogate model consisting of a set of 12 Gaussian process emulators:

$$\mathbf{f}_{emul} = (f_{emul,\,1},\, \dots,\, f_{emul,\,12}) \tag{7}$$

and used the emulator posterior mean to estimate the Sobol indices, namely:

$$\mathbb{E}[\mathbf{f}_{emul}(X)] \xmapsto{\mathcal{S}(\cdot)} S_i,\, S_{ij},\, S_T \tag{8}$$

In order to show that small uncertainties do not affect sensitivity indices estimates, we performed an additional example simulation in which we took into account the emulator posterior variance in the estimation of the SHAM rat model ejection fraction (EF) global sensitivities to model parameters. This was performed by evaluating the Saltelli estimator $\mathcal{S}$ at full emulator posterior predictions $\hat{Y}$, which correspond to values randomly sampled from a multivariate normal distribution with mean vector given by the specific emulator of EF feature ($f_{emul,\,3}$) posterior mean $\boldsymbol{\mu} = \mathbb{E}[f_{emul,\,3}(X)]$, and covariance matrix given by the same emulator posterior covariance matrix $\Sigma = \mathrm{Cov}[f_{emul,\,3}(X)]$, namely:

$$\mathcal{N}(\boldsymbol{\mu},\, \Sigma) \sim \hat{Y} \xmapsto{\mathcal{S}(\cdot)} S_i,\, S_{ij},\, S_T \tag{9}$$

We sampled $1,000$ emulator posterior predictions ($\hat{Y}_k$, for $k = 1, \dots, 1000$) and we compared the obtained sensitivity indices $(S_i,\, S_{ij},\, S_T)_k$ with the ones obtained when only the emulator posterior mean was used (equation (8)). In Figure 16, each sensitivity index' distributions with 2 STD are plotted against the single values obtained when the posterior mean only is run instead. We can see that the distributions' means are almost perfectly matching the single-valued effects for each sensitivity index. The similarities of the sensitivities is consistent with a limited impact of GPE uncertainties on GSA calculation.

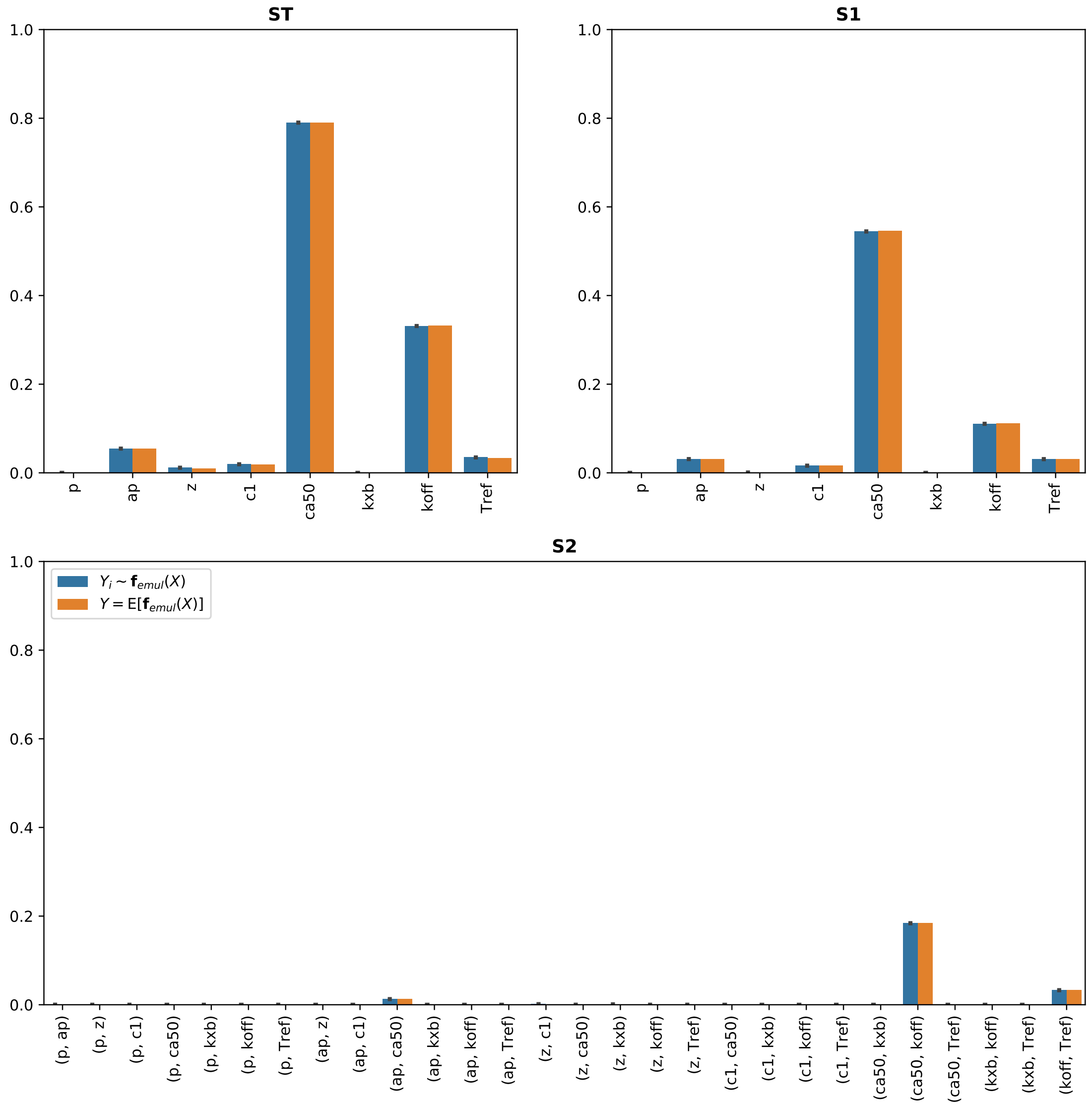

Figure 16: Ejection fraction global sensitivity to model parameters. $1,000$ predictions from the emulator posterior distribution are sampled, and first- and second-order and total effects (S_i, S_{ij}, S_T) are computed for each of them. The obtained indices distributions are plotted with $\sim 95\%$ confidence intervals (blue), compared with the sensitivity indices single values obtained when the emulator posterior mean only is used as a pointwise prediction in the sensitivity indices calculation (orange).

Modelling heart tissue mechanics

In this Section we provide a description of the employed mathematical model of heart tissue mechanics.

Tissue models of cardiac mechanics link cellular active contraction models through to whole organ pump function. We modelled mechanical properties of the cardiac tissue using non-linear solid mechanics. Like electrical properties, tissue mechanical properties depend on the orientation of cardiac tissue microstructure. Therefore, in the following description, tensors will be given in terms of a coordinate system locally aligned with the muscle fibre ($\mathbf{f}$), sheet ($\mathbf{s}$) and sheet-normal directions ($\mathbf{n}$) in the reference configuration, determining the orthonormal matrix $\mathbf{L} = [\mathbf{f}\,\mathbf{s}\,\mathbf{n}]$. In finite elastic deformation theory, the transformation from undeformed ($\mathbf{X}$) configuration to deformed ($\mathbf{x}$) configuration is described by means of the deformation gradient tensor:

$$\mathbf{F} = \frac{\partial \mathbf{x}}{\partial \mathbf{X}} \tag{10}$$

Large deformation mechanics equations enforce the stress equilibrium:

$$-\mathrm{Div}(\mathbf{F}\mathbf{T}) = 0 \tag{11}$$

where $\mathbf{T}$ is the second Piola-Kirchhoff stress tensor. The solution to the mechanics problem requires a description of the material behaviour known as the *constitutive law*, which gives the stress tensor $\mathbf{T}$ as a function of the Green strain tensor $\mathbf{E}$ and the Cauchy strain tensor $\mathbf{C}$ for the material. The strain tensor is given as a function of the deformation gradient tensor:

$$\mathbf{E} = \frac{1}{2}\,(\mathbf{C} - \mathbf{I}), \quad \mathbf{C} = \mathbf{F}^T\mathbf{F} \tag{12}$$

and is transformed to a fibre-aligned coordinate system using $\mathbf{E}^{\mathbf{fsn}} = \mathbf{L}^T\,\mathbf{E}\,\mathbf{L}$. The stress tensor $\mathbf{T}^{\mathbf{fsn}}$ is determined from the constitutive law, which is generally expressed as a strain energy function W:

$$\mathbf{T}^{\mathbf{fsn}} = \frac{\partial W}{\partial \mathbf{E}^{\mathbf{fsn}}} \tag{13}$$

The stress tensor $\mathbf{T}^{\mathbf{fsn}}$ is then transformed back using $\mathbf{L}\,\mathbf{T}^{\mathbf{fsn}}\,\mathbf{L}^T$. In this work, we used the transversely isotropic cardiac strain energy function proposed by Guccione et al. [52], combined with a Lagrange multiplier scheme with a compressibility penalty term [53, 54]:

$$W = W_g - p(J-1) + \frac{\kappa}{2}(J-1)^2 \tag{14}$$

where

$$W_g = \frac{1}{2}\,c_1\,(e^{Q(\mathbf{E})} - 1) \tag{15}$$

$$Q(\mathbf{E}) = c_2\,E_{\mathrm{ff}}^2 + c_3\,(E_{\mathrm{ss}}^2 + E_{\mathrm{nn}}^2 + 2\,E_{\mathrm{sn}}^2) + 2\,c_4\,(E_{\mathrm{fs}}^2 + E_{\mathrm{fn}}^2) \tag{16}$$

and where κ, c_1, c_2, c_3, $c_4 \in \mathbb{R}^+$, p is the hydrostatic term and $J = \det(\mathbf{F})$. The final second Piola-Kirchhoff stress tensor is obtained from the strain energy function by adding an active tension component T_a (calculated using the Land et al. [16] model of cellular contraction) along the fibre direction $\mathbf{f}$:

$$\mathbf{T} = \mathbf{L}\,\frac{\partial W}{\partial \mathbf{E}^{\mathbf{fsn}}}\,\mathbf{L}^T + T_a\,\mathbf{f}\mathbf{f}^T \tag{17}$$

$\mathbf{T}$ expresses the stresses in actively contracting incompressible cardiac tissue in terms of its strain and completes the set of equations required to model actively contracting cardiac tissue. The resulting system of equations is solved using the finite element method.

LV features extraction

In this Section we describe how specifically the 12 left ventricular (LV) features under study were extracted from the model output.

The multi-scale model of rat heart contraction is run for a specific input parameters set for 4 heart beats. The 12 key LV features are then extracted from the fourth beat's volume $V(t)$ and pressure $P(t)$ curves as follows:

$$\text{EDV} = \max_{t>0} V(t) \tag{18}$$

$$\text{ESV} = \min_{t>0} V(t) \tag{19}$$

$$\text{EF} = 100 \times \frac{\text{EDV} - \text{ESV}}{\text{EDV}} \tag{20}$$

$$\text{IVCT} = t_1 - t_0 \tag{21}$$

$$\text{ET} = t_2 - t_1 \tag{22}$$

$$\text{IVRT} = t_3 - t_2 \tag{23}$$

$$\text{Tdiast} = t_4 - t_2 \tag{24}$$

$$\text{PeakP} = \max_{t>0} P(t) \tag{25}$$

$$\text{Tpeak} = \arg\max_{t>0} P(t) \quad (= P(t_5)) \tag{26}$$

$$\text{ESP} = P(t_2) \tag{27}$$

$$\text{maxdP} = \max_{t>0} \frac{dP(t)}{dt} \tag{28}$$

$$\text{mindP} = \min_{t>0} \frac{dP(t)}{dt} \tag{29}$$

where t_0, t_1, t_2, t_3, t_4, t_5 are all positive time points, shown in Figure 17.

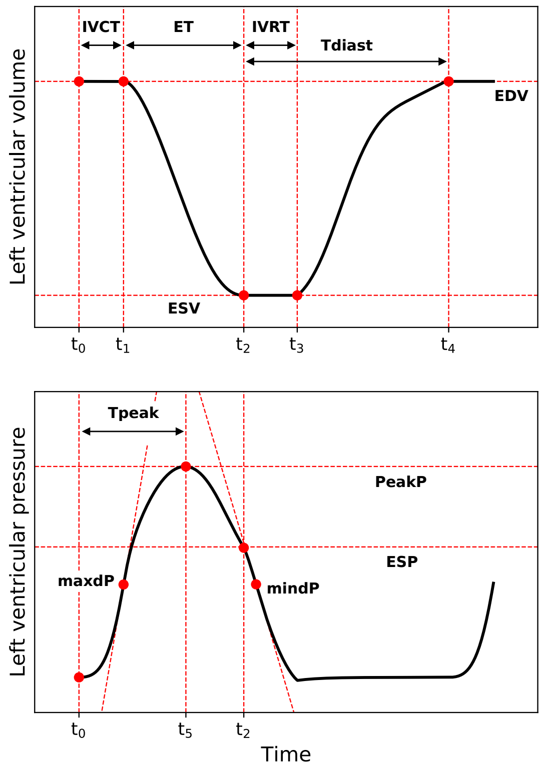

Figure 17: LV features (EDV, ESV, EF, IVCT, ET, IVRT, Tdiast, PeakP, Tpeak, ESP, maxdP, mindP) extracted from left ventricular volume and pressure transients.

References

[1] Rasmussen CE, Williams CKI. Gaussian Processes for Machine Learning (Adaptive Computation and Machine Learning). The MIT Press; 2005.

[2] Coveney S, Clayton RH. Fitting two human atrial cell models to experimental data using Bayesian history matching. Prog Biophys Mol Biol. 2018;139:43–58.

[3] Vernon I, Goldsteiny M, Bowerz RG. Galaxy formation: A Bayesian uncertainty analysis. Bayesian Anal. 2010;5(4):619–670.

[4] Vernon I, Liu J, Goldstein M, Rowe J, Topping J, Lindsey K. Bayesian uncertainty analysis for complex systems biology models: emulation, global parameter searches and evaluation of gene functions. BMC Syst Biol. 2018;12(1):1.

[5] Pukelsheim F. The Three Sigma Rule. The American Statistician. 1994;48(2):88–91. Available from: https://amstat.tandfonline.com/doi/abs/10.1080/00031305.1994.10476030.

[6] Røe ÅT, Aronsen JM, Skårdal K, Hamdani N, Linke WA, Danielsen HE, et al. Increased passive stiffness promotes diastolic dysfunction despite improved Ca2+ handling during left ventricular concentric hypertrophy. Cardiovasc Res. 2017;113(10):1161–1172.

[7] Németh BT, Mátyás C, Oláh A, Lux Á, Hidi L, Ruppert M, et al. Cinaciguat prevents the development of pathologic hypertrophy in a rat model of left ventricular pressure overload. Sci Rep. 2016;6.

[8] Sato F, Isoyama S, Takishima T. Normalization of impaired coronary circulation in hypertrophied rat hearts. Hypertension. 1990;16(1):26–34.

[9] Schunkert H, Weinberg EO, Bruckschlegel G, Riegger AJ, Lorell BH. Alteration of growth responses in established cardiac pressure overload hypertrophy in rats with aortic banding. J Clin Invest. 1995;96(6):2768–2774.

[10] Loot A. Therapeutic perspectives of Angiotensin-(1-7) in heart failure. University of Groningen; 2005.

[11] Liu J, Han P, Xiao Y, Liu J, James Kang Y, Jm L, et al. A novel knot method for individually measurable aortic constriction in rats. Am J Physiol Hear Circ Physiol. 2014;307:987–995.

[12] Ku HC, Su MJ. DPP4 deficiency preserved cardiac function in abdominal aortic banding rats. PLoS One. 2014;9(1).

[13] Ruppert M, Korkmaz-Icöz S, Loganathan S, Jiang W, Lehmann L, Oláh A, et al. Pressure-volume analysis reveals characteristic sex-related differences in cardiac function in a rat model of aortic banding-induced myocardial hypertrophy. Am J Physiol Hear Circ Physiol. 2018;315:502–511.

[14] Schunkert H, Dzau VJ, Tang SS, Hirsch AT, Apstein CS, Lorell BH. Increased rat cardiac angiotensin converting enzyme activity and mRNA expression in pressure overload left ventricular hypertrophy. Effects on coronary resistance, contractility, and relaxation. J Clin Invest. 1990;86(6):1913–1920.

[15] Ruppert M, Korkmaz-Icöz S, Li S, Németh BT, Hegedús P, Brlecic P, et al. Myocardial reverse remodeling after pressure unloading is associated with maintained cardiac mechanoenergetics in a rat model of left ventricular hypertrophy. Am J Physiol Hear Circ Physiol. 2016;311:592–603.

[16] Land S, Niederer SA, Aronsen JM, Espe EKS, Zhang L, Louch WE, et al. An analysis of deformation-dependent electromechanical coupling in the mouse heart. J Physiol. 2012;590(18):4553–4569.

[17] Lewalle A, Land S, Carruth E, Frank LR, Lamata P, Omens JH, et al. Decreasing compensatory ability of concentric ventricular hypertrophy in aortic-banded rat hearts. Front Physiol. 2018;9(FEB).

[18] Kovács A, Oláh A, Lux Á, Mátyás C, Németh T, Kellermayer D, et al. Strain and strain rate by speckle-tracking echocardiography correlate with pressure-volume loop-derived contractility indices in a rat model of athlete's heart. J Physiol Hear Circ Physiol. 2015;308:743–748.

[19] Lee K, Hwang HJ, Kim OS, Oh YJ. Assessment of dexmedetomidine effects on left ventricular function using pressure–volume loops in rats. J Anesth. 2017;31(1):18–24.

[20] Oláh A, Németh BT, Mátyás C, Horváth EM, Hidi L, Birtalan E, et al. Cardiac effects of acute exhaustive exercise in a rat model. Int J Cardiol. 2015;182(C):258–266.

[21] Toledo C, Andrade DC, Lucero C, Arce-Alvarez A, Díaz HS, Aliaga V, et al. Cardiac diastolic and autonomic dysfunction are aggravated by central chemoreflex activation in heart failure with preserved ejection fraction rats. J Physiol. 2017;595(8):2479–2495.

[22] Kobayashi S, Yano M, Kohno M, Obayashi M, Hisamatsu Y, Ryoke T, et al. Influence of aortic impedance on the development of pressure-overload left ventricular hypertrophy in rats. Circulation. 1996;94(12):3362–8.

[23] Levy BI, Michel JB, Salzmann JL, Azizi M, Poitevin P, Safar M, et al. Effects of chronic inhibition of converting enzyme on mechanical and structural properties of arteries in rat renovascular hypertension. Circ Res. 1988;63(1):227–39.

[24] Zuckerman BD, Yin FC. Aortic impedance and compliance in hypertensive rats. Am J Physiol Circ Physiol. 1989;257(2):H553–H562.

[25] Lin YT, Tseng YZ, Chang KC. Aminoguanidine Prevents Fructose-Induced Arterial Stiffening in Wistar Rats: Aortic Impedance Analysis. Exp Biol Med. 2004;229(10):1038–1045.

[26] Yin FC, Spurgeon HA, Weisfeldt ML, Lakatta EG. Mechanical properties of myocardium from hypertrophied rat hearts. A comparison between hypertrophy induced by senescence and by aortic banding. Circ Res. 1980;46(2):292–300.

[27] Ioannou CV, Morel DR, Katsamouris AN, Katranitsa S, Startchik I, Kalangos A, et al. Left ventricular hypertrophy induced by reduced aortic compliance. J Vasc Res. 2009;46(5):417–425.

[28] Chang RW, Chang CY, Wu MS, Yu HY, Luo JM, Chen YS, et al. Systolic aortic pressure-time area is a useful index describing arterial wave properties in rats with diabetes. Sci Rep. 2015;5.

[29] Westerhof N, Elzinga G. Normalized input impedance and arterial decay time over heart period are independent of animal size. Am J Physiol Integr Comp Physiol. 1991;261(1):R126–R133.

[30] Nordbø Ø, Lamata P, Land S, Niederer S, Aronsen JM, Louch WE, et al. A computational pipeline for quantification of mouse myocardial stiffness parameters. Comput Biol Med. 2014;53:65–75.

[31] Niederer SA, Smith NP. The Role of the Frank–Starling Law in the Transduction of Cellular Work to Whole Organ Pump Function: A Computational Modeling Analysis. PLoS Comput Biol. 2009;5(4):e1000371.

[32] Omens JH, MacKenna DA, McCulloch AD. Measurement of strain and analysis of stress in resting rat left ventricular myocardium. J Biomech. 1993;26(6):665–676.

[33] Niederer SA, Hunter PJ, Smith NP. A quantitative analysis of cardiac myocyte relaxation: A simulation study. Biophys J. 2006;90(5):1697–1722.

[34] Gao WD, Backx PH, Azan-Backx M, Marban E. Myofilament Ca2+ sensitivity in intact versus skinned rat ventricular muscle. Circ Res. 1994;74(3):408–15.

[35] Gao WD, Atar D, Backx PH, Marban E. Relationship between intracellular calcium and contractile force in stunned myocardium. Direct evidence for decreased myofilament Ca2+ responsiveness and altered diastolic function in intact ventricular muscle. Circ Res. 1995;76(6):1036–48.

[36] Backx PH, Gao WD, Azan-Backx MD, Marban E. The relationship between contractile force and intracellular [Ca2+] in intact rat cardiac trabeculae. J Gen Physiol. 1995;105(1):1–19.

[37] Blanchard E, Seidman C, Seidman JG, LeWinter M, Maughan D. Altered crossbridge kinetics in the alphaMHC403/+ mouse model of familial hypertrophic cardiomyopathy. Circ Res. 1999;84(4):475–83.

[38] Stull LB, Leppo MK, Marbán E, Janssen PML. Physiological Determinants of Contractile Force Generation and Calcium Handling in Mouse Myocardium. J Mol Cell Cardiol. 2002;34(10):1367–1376.

[39] Rosenfeld SS, Taylor EW. Kinetic studies of calcium binding to regulatory complexes from skeletal muscle. J Biol Chem. 1985;260(1):252–61.

[40] Tikunova SB, Davis JP. Designing calcium-sensitizing mutations in the regulatory domain of cardiac troponin C. J Biol Chem. 2004;279(34):35341–35352.

[41] Davis JP, Norman C, Kobayashi T, Solaro RJ, Swartz DR, Tikunova SB. Effects of thin and thick filament proteins on calcium binding and exchange with cardiac troponin C. Biophys J. 2007;92(9):3195–3206.

[42] Stuyvers BD, McCulloch AD, Guo J, Duff HJ, Ter Keurs HEDJ. Effect of stimulation rate, sarcomere length and Ca2+ on force generation by mouse cardiac muscle. J Physiol. 2002;544(3):817–830.

[43] Palmer BM, Fishbaugher DE, Schmitt JP, Wang Y, Alpert NR, Seidman CE, et al. Differential cross-bridge kinetics of FHC myosin mutations R403Q and R453C in heterozygous mouse myocardium. Am J Physiol Hear Circ Physiol. 2004;287:91–99.

[44] Kreutziger KL, Piroddi N, McMichael JT, Tesi C, Poggesi C, Regnier M. Calcium binding kinetics of troponin C strongly modulate cooperative activation and tension kinetics in cardiac muscle. J Mol Cell Cardiol. 2011;50(1):165–174.

[45] Rice JJ, Wang F, Bers DM, De Tombe PP. Approximate model of cooperative activation and crossbridge cycling in cardiac muscle using ordinary differential equations. Biophys J. 2008;95(5):2368–2390.

[46] Bovendeerd PHM, Kroon W, Delhaas T. Determinants of left ventricular shear strain. Am J Physiol Circ Physiol. 2009;297(3):H1058–H1068.

[47] Oakley JE, O'Hagan A. Probabilistic Sensitivity Analysis of Complex Models: A Bayesian Approach. Journal of the Royal Statistical Society Series B (Statistical Methodology). 2004;66(3):751–769.

[48] Noè U, Lazarus A, Gao H, Davies V, Macdonald B, Mangion K, et al. Gaussian process emulation to accelerate parameter estimation in a mechanical model of the left ventricle: a critical step towards clinical end-user relevance. Journal of The Royal Society Interface. 2019;16(156):20190114.

[49] Parker K, Ruggiero P, Serafin KA, Hill DF. Emulation as an approach for rapid estuarine modeling. Coastal Engineering. 2019;150:79 – 93. Available from: `http://www.sciencedirect.com/science/article/pii/S0378383918305532`.

[50] Salter JM, Williamson D. A comparison of statistical emulation methodologies for multi-wave calibration of environmental models. Environmetrics. 2016;27(8):507–523.

[51] Saltelli A, Annoni P, Azzini I, Campolongo F, Ratto M, Tarantola S. Variance based sensitivity analysis of model output. Design and estimator for the total sensitivity index. Computer Physics Communications. 2010;181(2):259–270.

[52] Guccione JM, McCulloch AD, Waldman LK. Passive material properties of intact ventricular myocardium determined from a cylindrical model. J Biomech Eng. 1991;113(1):42–55.

[53] Land S, Niederer SA, Smith NP. Efficient computational methods for strongly coupled cardiac electromechanics. IEEE Trans Biomed Eng. 2012;59(5):1219–1228.

[54] Land S, Niederer SA, Lamata P, Smith NP. Improving the stability of cardiac mechanical simulations. IEEE Trans Biomed Eng. 2015;62(3):939–947.

[55] Camacho P, Fan H, Liu Z, He JQ. Small mammalian animal models of heart disease. Am J Cardiovasc Dis. 2016;6(3):70–80.

[56] Patten RD, Hall-Porter MR. Small Animal Models of Heart Failure. Circ Hear Fail. 2009;2(2):138–144.

[57] Westerhof N, Elzinga G, Sipkema P. An artificial arterial system for pumping hearts. J Appl Physiol. 1971 Nov;31(5):776–781.

[58] Henkel DM, Redfield MM, Weston SA, Gerber Y, Roger VL. Death in Heart Failure. Circ Hear Fail. 2008;1(2):91–97.

[59] Owan TE, Hodge DO, Herges RM, Jacobsen SJ, Roger VL, Redfield MM. Trends in Prevalence and Outcome of Heart Failure with Preserved Ejection Fraction. N Engl J Med. 2006;355(3):251–259.

[60] Chang ETY, Strong M, Clayton RH. Bayesian Sensitivity Analysis of a Cardiac Cell Model Using a Gaussian Process Emulator. PLOS ONE. 2015 06;10(6):1–20.

[61] Lamata P, Roy I, Blazevic B, Crozier A, Land S, Niederer SA, et al. Quality Metrics for High Order Meshes: Analysis of the Mechanical Simulation of the Heart Beat. IEEE Transactions on Medical Imaging. 2013 Jan;32(1):130–138.

[62] Lamata P, Niederer S, Nordsletten D, Barber DC, Roy I, Hose DR, et al. An accurate, fast and robust method to generate patient-specific cubic Hermite meshes. Med Image Anal. 2011;15(6):801–813.

[63] Lamata P, Sinclair M, Kerfoot E, Lee A, Crozier A, Blazevic B, et al. An automatic service for the personalization of ventricular cardiac meshes. J R Soc Interface. 2014;11(91).

[64] Gattoni S, Røe ÅT, Frisk M, Louch WE, Niederer SA, Smith NP. The calcium–frequency response in the rat ventricular myocyte: an experimental and modelling study. J Physiol. 2016;594(15):4193–4224.

[65] Land S, Gurev V, Arens S, Augustin CM, Baron L, Blake R, et al. Verification of cardiac mechanics software: benchmark problems and solutions for testing active and passive material behaviour. Proceedings of the Royal Society A: Mathematical, Physical and Engineering Sciences. 2015;471(2184):20150641.

[66] Conceição G, Heinonen I, Lourenço AP, Duncker DJ, Falcão-Pires I. Animal models of heart failure with preserved ejection fraction. Netherlands Hear J. 2016;24(4):275–286.

[67] Andrianakis I, Vernon IR, McCreesh N, McKinley TJ, Oakley JE, Nsubuga RN, et al. Bayesian History Matching of Complex Infectious Disease Models Using Emulation: A Tutorial and a Case Study on HIV in Uganda. PLoS Comput Biol. 2015;11(1):e1003968.

[68] Houser SR, Margulies KB, Murphy AM, Spinale FG, Francis GS, Prabhu SD, et al. Animal Models of Heart Failure. Circ Res. 2012;111(1):131–150.

[69] Adelborg K, Szépligeti S, Sundbøll J, Horváth-Puhó E, Henderson VW, Ording A, et al. Risk of Stroke in Patients With Heart Failure. Stroke. 2017;48(5):1161–1168.

[70] Gattoni S, Røe ÅT, Aronsen JM, Sjaastad I, Louch WE, Smith NP, et al. Compensatory and decompensatory alterations in cardiomyocyte Ca2+ dynamics in hearts with diastolic dysfunction following aortic banding. J Physiol. 2017;595(12):3867–3889.

[71] Bishop CM. Pattern Recognition and Machine Learning (Information Science and Statistics). Berlin, Heidelberg: Springer-Verlag; 2006.

[72] Bhf.org.uk. (2019). Heart and Circulatory Diseases Statistics 2019;. Accessed 7 Oct. 2019. `https://www.bhf.org.uk/what-we-do/our-research/heart-statistics/heart-statistics-publications/`.

[73] Sobol' IM. Global sensitivity indices for nonlinear mathematical models and their Monte Carlo estimates. Math Comput Simulat. 2001;55(1):271–280.

[74] Herman J, Usher W. SALib: An open-source Python library for Sensitivity Analysis. The Journal of Open Source Software. 2017;2(9).

[75] Niederer SA, Campbell KS, Campbell SG. A short history of the development of mathematical models of cardiac mechanics. J Mol Cell Cardiol. 2019;127:11–19.

[76] Loewe A, Wilhelms M, Schmid J, Krause MJ, Fischer F, Thomas D, et al. Parameter Estimation of Ion Current Formulations Requires Hybrid Optimization Approach to Be Both Accurate and Reliable;.

[77] Dokos S, Lovell NH. Parameter estimation in cardiac ionic models. Prog Biophys Mol Biol. 2004;85(2-3):407–431.

[78] Groenendaal W, Ortega FA, Kherlopian AR, Zygmunt AC, Krogh-Madsen T, Christini DJ. Cell-specific cardiac electrophysiology models. PLoS Comput Biol. 2015 Apr;11(4):e1004242.

[79] Sarkar AX, Sobie EA. Regression analysis for constraining free parameters in electrophysiological models of cardiac cells. PLoS Comput Biol. 2010 Sep;6(9):e1000914.

[80] Johnstone RH, Chang ETY, Bardenet R, de Boer TP, Gavaghan DJ, Pathmanathan P, et al. Uncertainty and variability in models of the cardiac action potential: Can we build trustworthy models? J Mol Cell Cardiol. 2016 07;96:49–62.

[81] Wang VY, Lam HI, Ennis DB, Cowan BR, Young AA, Nash MP. Modelling passive diastolic mechanics with quantitative MRI of cardiac structure and function. Med Image Anal. 2009;13(5):773–784. Includes Special Section on the 12th International Conference on Medical Imaging and Computer Assisted Intervention.

[82] Xi J, Lamata P, Niederer S, Land S, Shi W, Zhuang X, et al. The estimation of patient-specific cardiac diastolic functions from clinical measurements. Med Image Anal. 2013;17(2):133–146.

[83] Pathmanathan P, Gray R. Ensuring reliability of safety-critical clinical applications of computational cardiac models. Front Physiol. 2013;4:358.

[84] Sher AA, Wang K, Wathen A, Maybank PJ, Mirams GR, Abramson D, et al. A local sensitivity analysis method for developing biological models with identifiable parameters: Application to cardiac ionic channel modelling. Future Generation Computer Systems. 2013;29(2):591–598.

[85] Pathmanathan P, Cordeiro JM, Gray RA. Comprehensive Uncertainty Quantification and Sensitivity Analysis for Cardiac Action Potential Models. Frontiers in Physiology. 2019;10:721.

[86] Assessing Credibility of Computational Modeling through Verification & Validation;. Available from: https://www.asme.org/codes-standards/find-codes-standards/v-v-40-assessing-credibility-computational-modeling-verification-validation-application-medi

[87] Holzapfel GA, Ogden RW. Constitutive modelling of passive myocardium: a structurally based framework for material characterization. Philos Trans A Math Phys Eng Sci. 2009 09;367(1902):3445–3475.

[88] Conti S, Gosling JP, Oakley JE, O'Hagan A. Gaussian process emulation of dynamic computer codes. Biometrika. 2009 06;96(3):663–676.
