# Supplementary figures and images for: Predicting left ventricular contractile function via Gaussian process emulation in aortic-banded rats
Source: Philos Trans A Math Phys Eng Sci. 2020 May 25;378(2173):20190334. doi: 10.1098/rsta.2019.0334 (PMC7287330; doi:10.1098/rsta.2019.0334)

**(a)**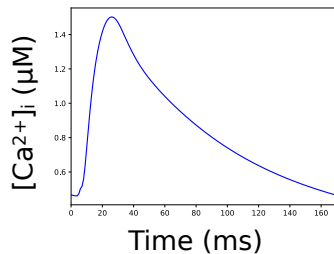**(b)**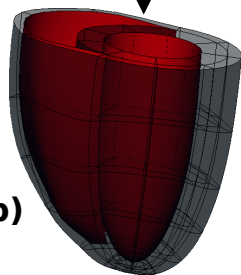LVV ( $\mu L$ )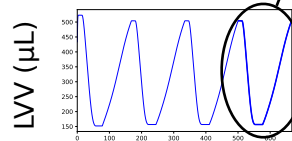

LVP (kPa)

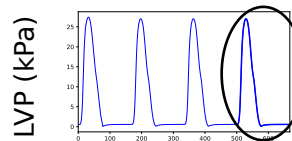**(c1)**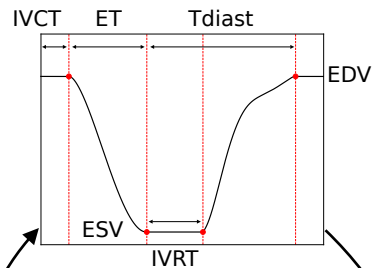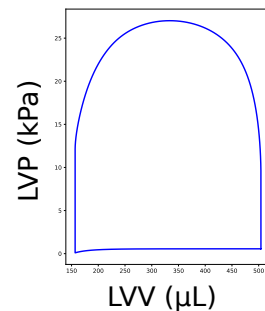**(c2)**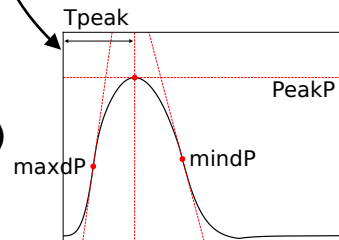**d =**

EDV  
ESV  
EF  
IVCT  
ET  
IVRT  
Tdiast  
PeakP  
Tpeak  
ESP  
maxdP  
mindP

Supplement: text_figures_tables.zip [file rsta20190334supp2.zip › rsta-2019-0334-File003/text_figures_tables/figures/1.pdf]

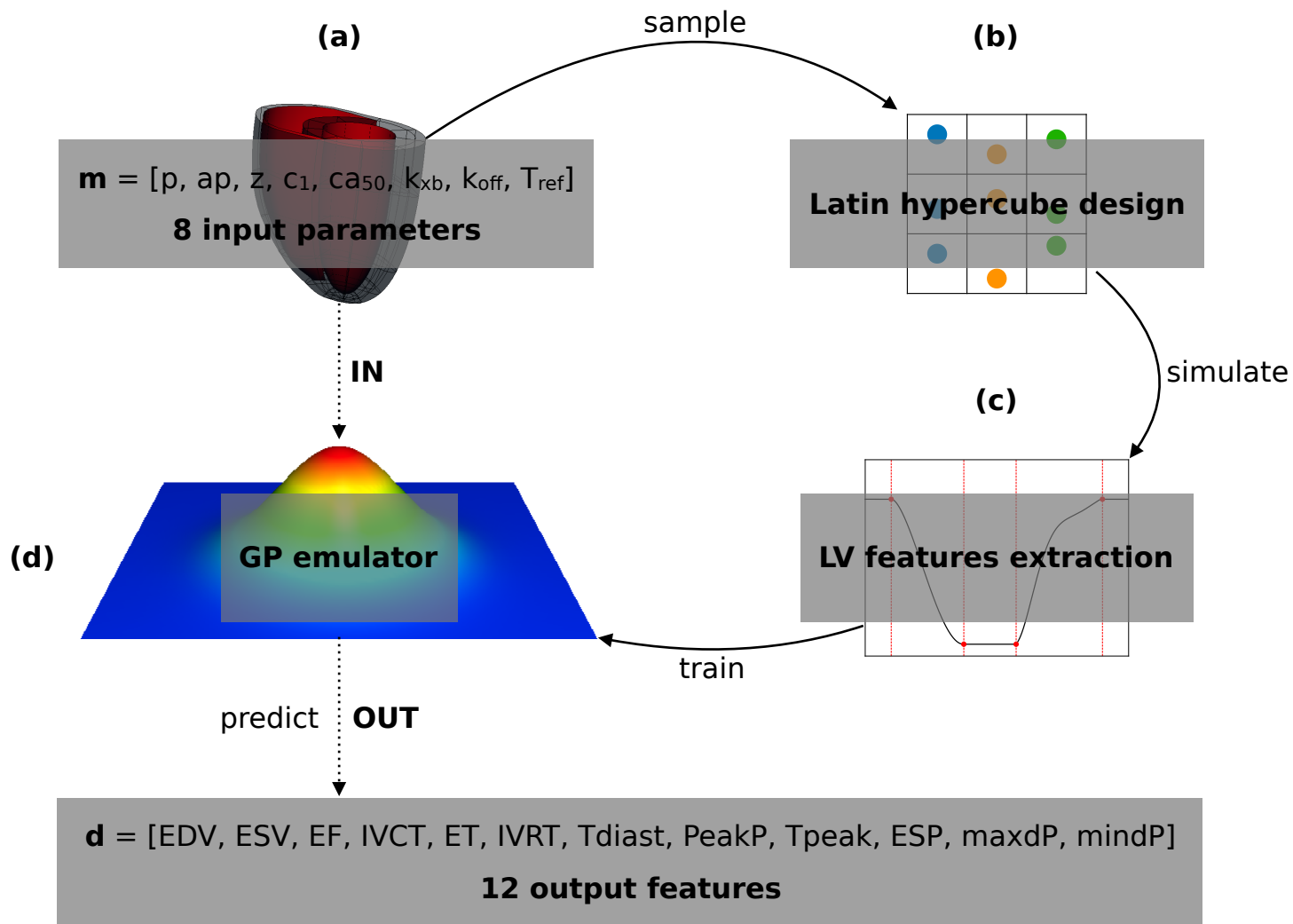

Supplement: text_figures_tables.zip [file rsta20190334supp2.zip › rsta-2019-0334-File003/text_figures_tables/figures/2.pdf]

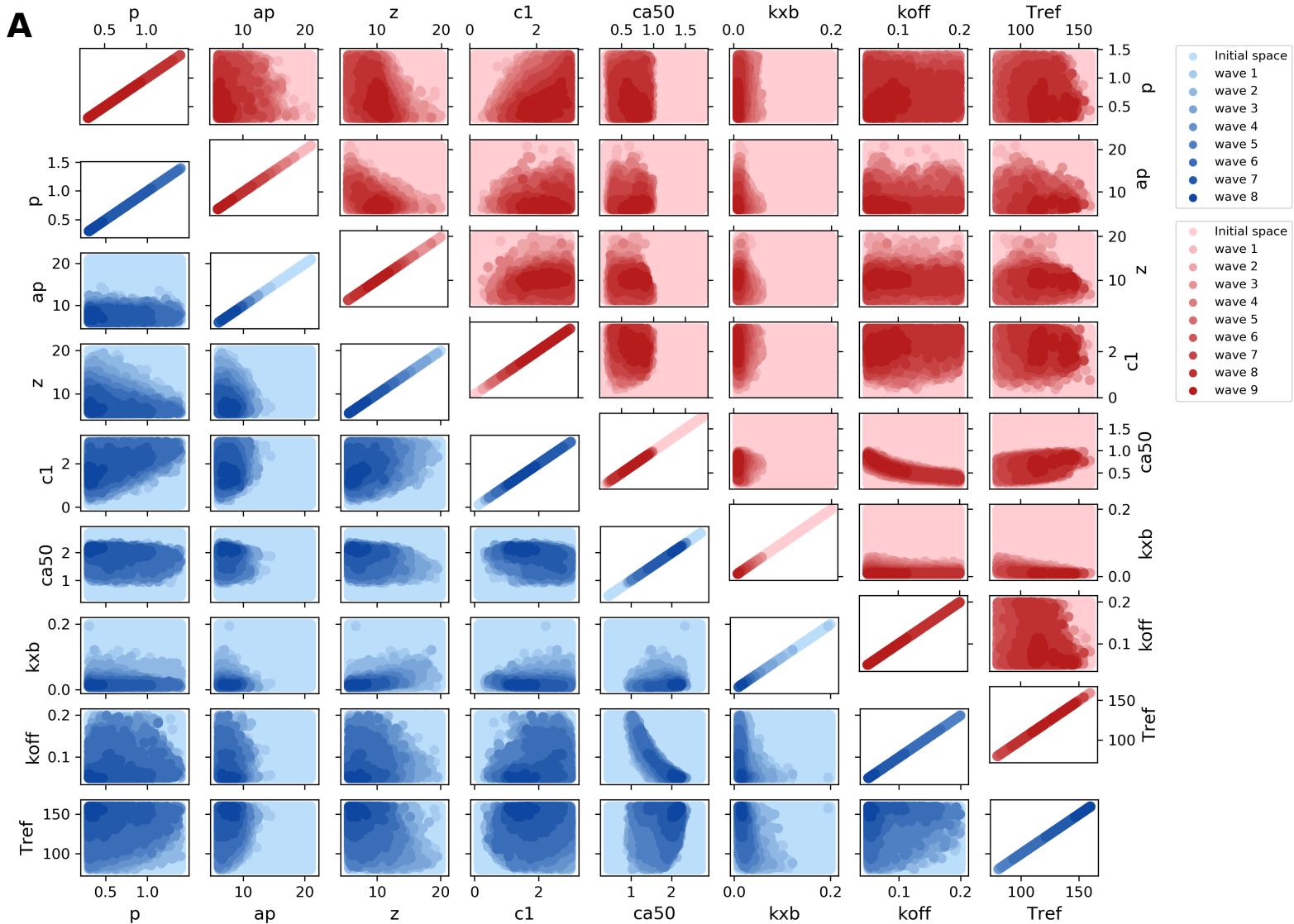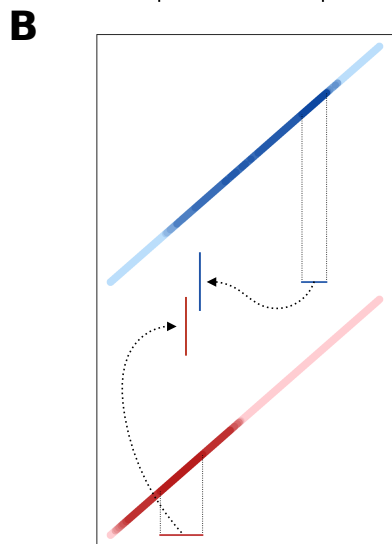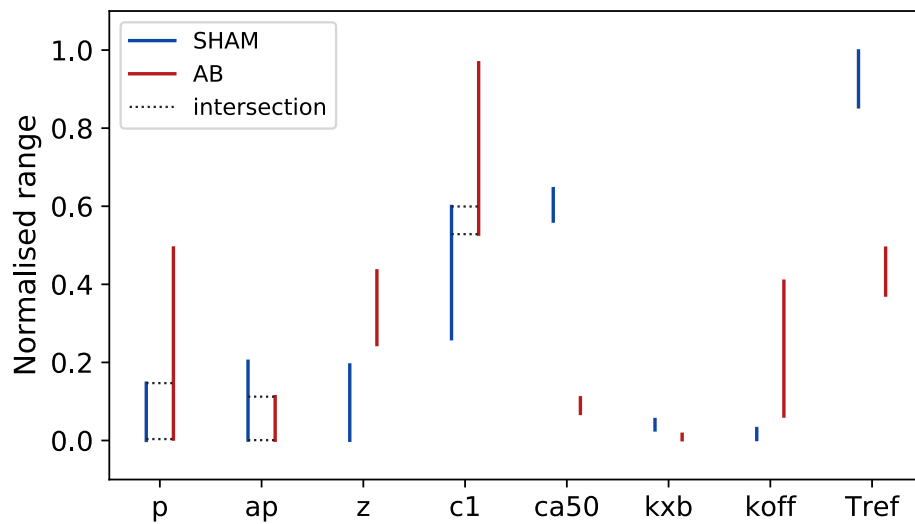

Supplement: text_figures_tables.zip [file rsta20190334supp2.zip › rsta-2019-0334-File003/text_figures_tables/figures/3.pdf]

**A.1**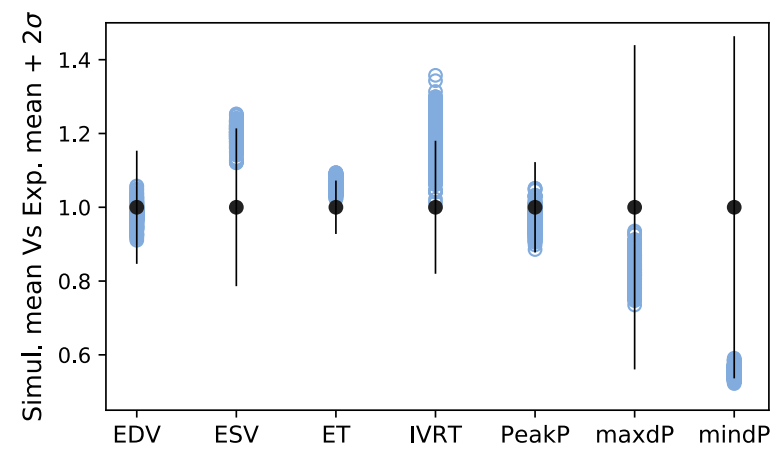**B.1**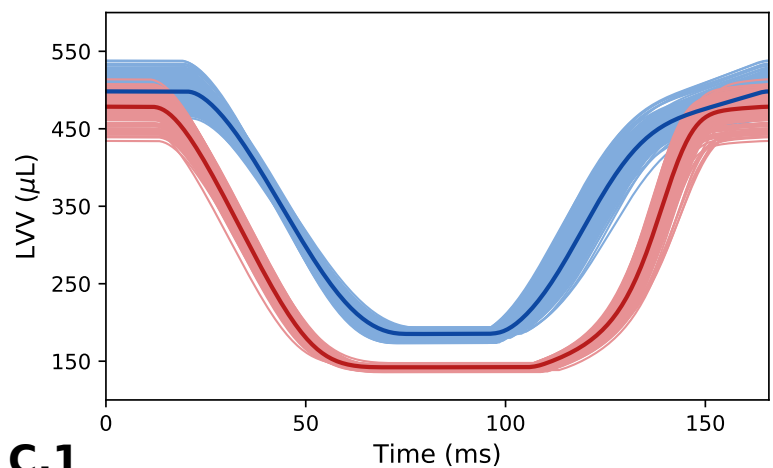**C.1**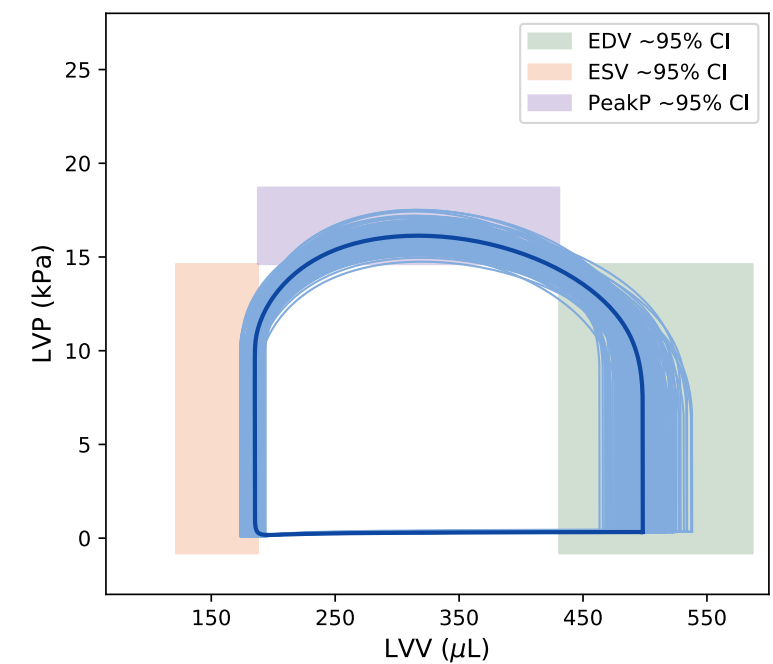**A.1**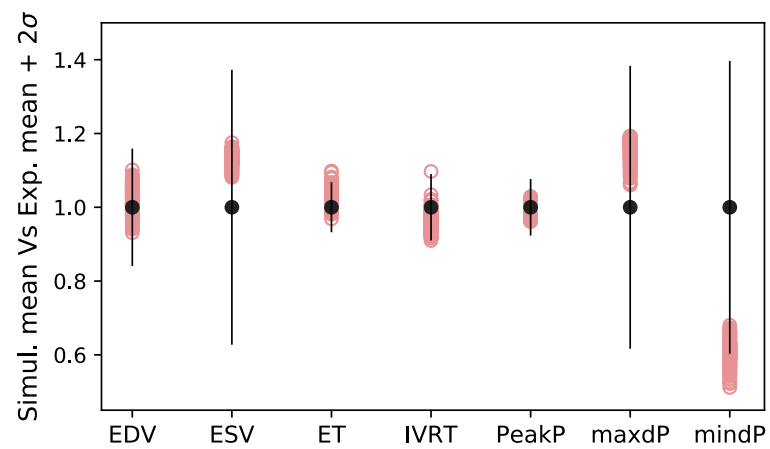**B.2**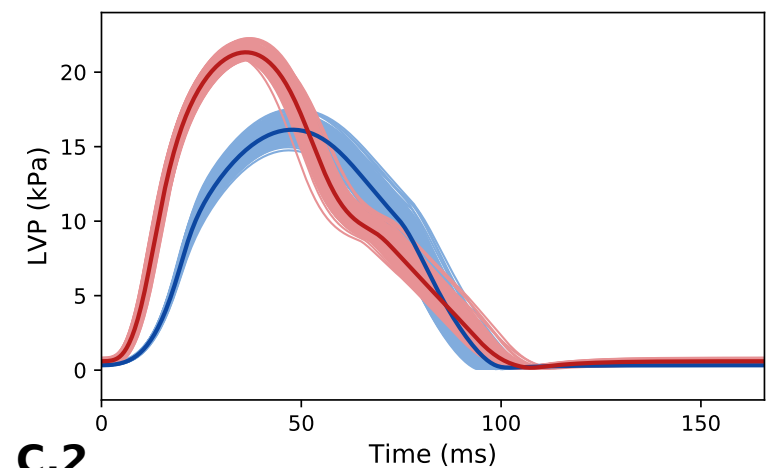**C.2**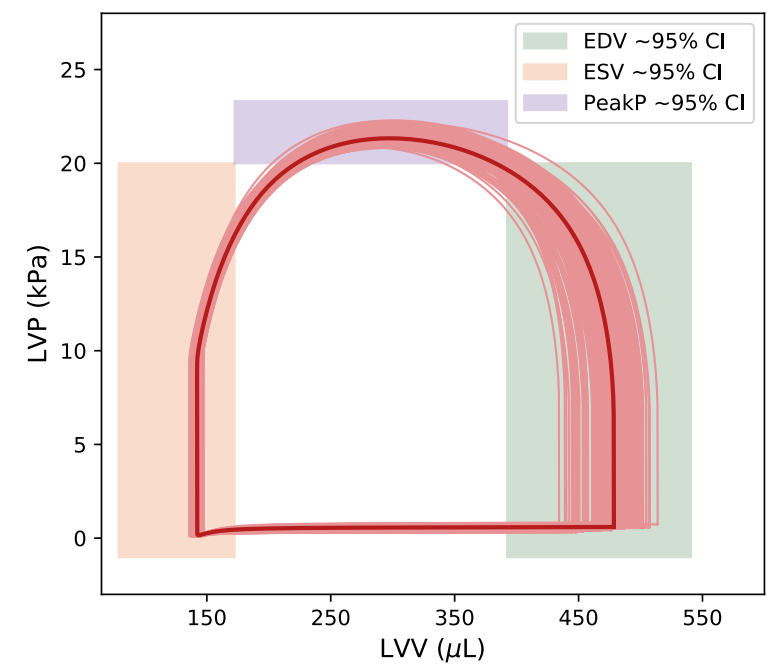

Supplement: text_figures_tables.zip [file rsta20190334supp2.zip › rsta-2019-0334-File003/text_figures_tables/figures/4.pdf]

**A**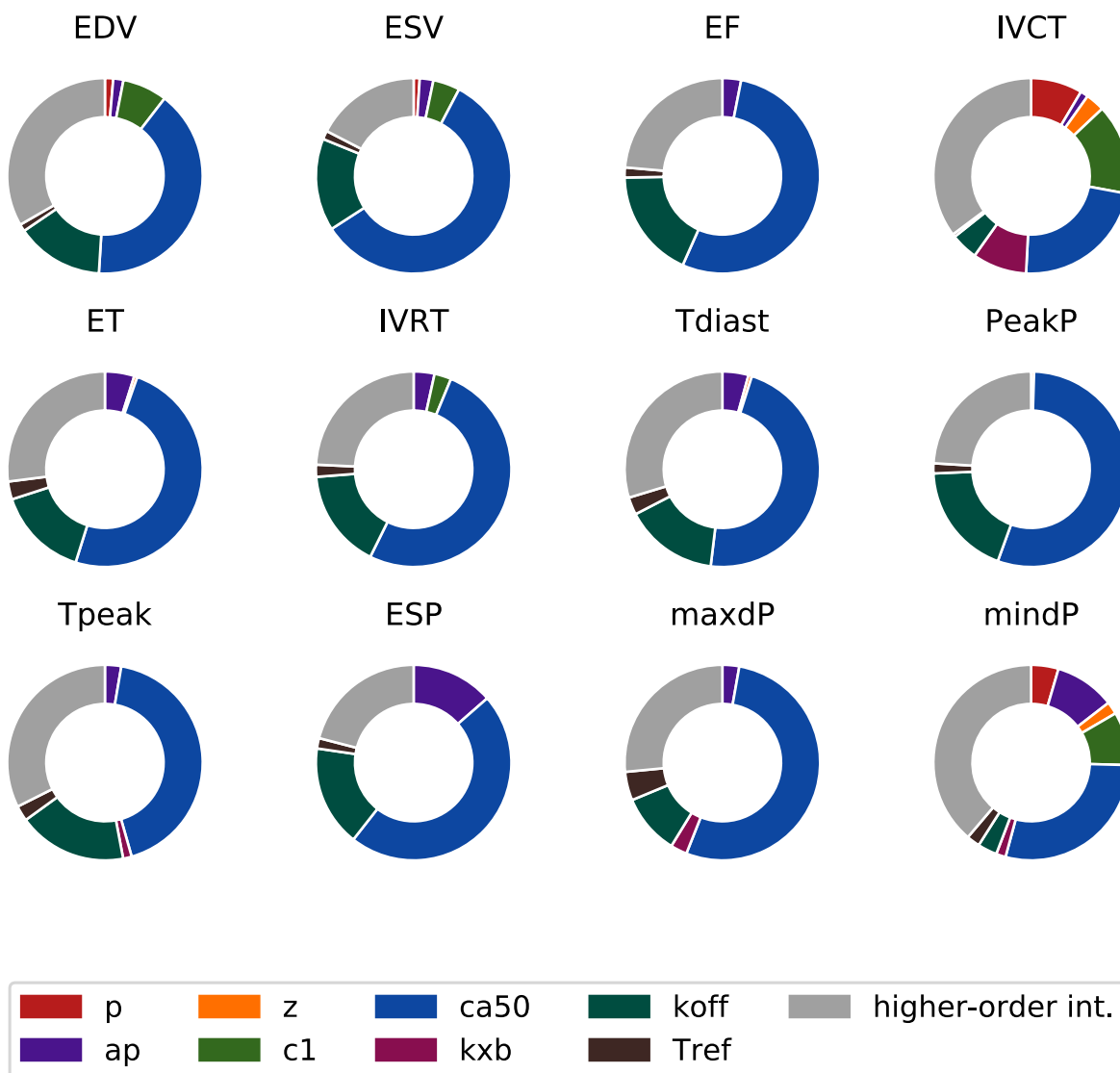**B**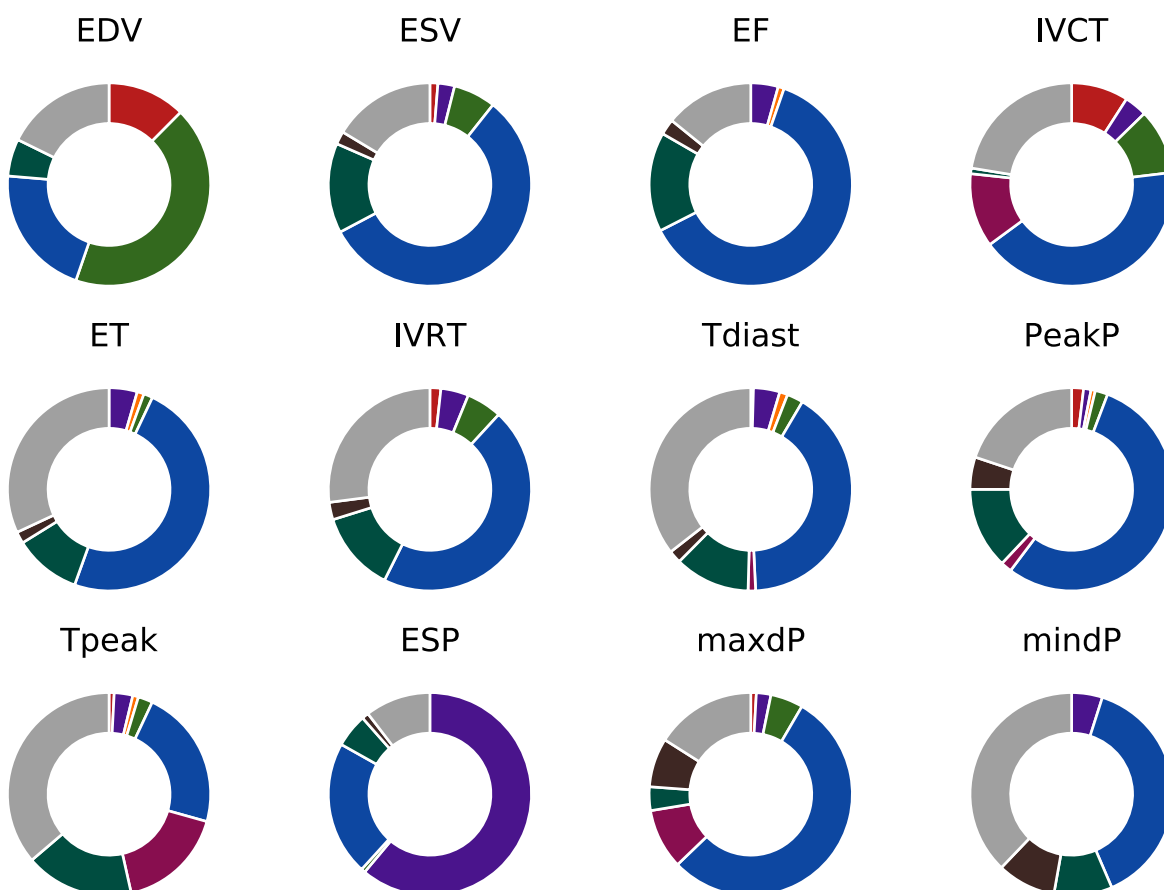

Supplement: text_figures_tables.zip [file rsta20190334supp2.zip › rsta-2019-0334-File003/text_figures_tables/figures/5.pdf]
